# The CEBPB^+^ glioblastoma subcluster specifically drives the formation of M2 tumor-associated macrophages to promote malignancy growth

**DOI:** 10.7150/thno.93473

**Published:** 2024-07-02

**Authors:** Yongchang Yang, Xingyu Jin, Yang Xie, Chunlan Ning, Yiding Ai, Haotian Wei, Xing Xu, Xianglian Ge, Tailong Yi, Qiang Huang, Xuejun Yang, Tao Jiang, Xiaoguang Wang, Yingzhe Piao, Xun Jin

**Affiliations:** 1 Department of Biochemistry and Molecular Biology, Tianjin Medical University Cancer Institute and Hospital, National Clinical Research Center for Cancer, Key Laboratory of Cancer Prevention and Therapy, Tianjin, Tianjin's Clinical Research Center for Cancer, Tianjin 300060, China.; 2 Tianjin Medical University, Tianjin 300060, China.; 3 Department of Neurosurgery, Beijing Tsinghua Changgung Hospital, Tsinghua University, Beijing, People's Republic of China.; 4 Department of Neurosurgery, Tianjin Medical University General Hospital, Tianjin, 300052, China.; 5 Beijing Neurosurgical Institute, Capital Medical University, Beijing, China.; 6 Department of Neuro-Oncology and Neurosurgery, Tianjin Medical University Cancer Institute and Hospital, National Clinical Research Center for Cancer, Key Laboratory of Cancer Prevention and Therapy, Tianjin China.

**Keywords:** Glioblastoma microenvironment, Single cell sequencing, Spatial transcriptome, CEBPB^+^ glioblastoma subcluster, M2 Tumor-associated macrophages, SPP1-Integrin αvβ1-Akt axis

## Abstract

**Rationale:** The heterogeneity of tumor cells within the glioblastoma (GBM) microenvironment presents a complex challenge in curbing GBM progression. Understanding the specific mechanisms of interaction between different GBM cell subclusters and non-tumor cells is crucial.

**Methods:** In this study, we utilized a comprehensive approach integrating glioma single-cell and spatial transcriptomics. This allowed us to examine the molecular interactions and spatial localization within GBM, focusing on a specific tumor cell subcluster, GBM subcluster 6, and M2-type tumor-associated macrophages (M2 TAMs).

**Results:** Our analysis revealed a significant correlation between a specific tumor cell subcluster, GBM cluster 6, and M2-type TAMs. Further in vitro and in vivo experiments demonstrated the specific regulatory role of the CEBPB transcriptional network in GBM subcluster 6, which governs its tumorigenicity, recruitment of M2 TAMs, and polarization. This regulation involves molecules such as MCP1 for macrophage recruitment and the SPP1-Integrin αvβ1-Akt signaling pathway for M2 polarization.

**Conclusion:** Our findings not only deepen our understanding of the formation of M2 TAMs, particularly highlighting the differential roles played by heterogeneous cells within GBM in this process, but also provided new insights for effectively controlling the malignant progression of GBM.

## Introduction

Glioblastoma (GBM) is the most aggressive primary brain tumor, accounting for approximately 28% of all brain tumors, but it is responsible for the majority of deaths 1. Despite the utilization of surgery, radiation, and chemotherapy as part of a comprehensive treatment approach, the median survival rate for patients with GBM remains under 15 months 2, 3. GBM exhibits a remarkable degree of heterogeneity, which is evident in several aspects: (1) Genetic heterogeneity, characterized by the presence of multiple genetically distinct subclones within individual GBM tumors 4. An unsupervised analysis of the transcriptome has identified three subtypes—termed classical (CL), mesenchymal (MES), and proneural (PN)—which are closely linked with genetic aberrations 5. The MES subtype is associated with a poorer prognosis and is implicated in disease recurrence and treatment resistance, making it a key factor in the malignant progression of GBM 5-7. (2) Epigenetic heterogeneity, demonstrated by malignant GBM cells mimicking developmental cellular hierarchies and adopting a diverse range of epigenetically determined transcriptional states 8, 9 and (3) Environmental heterogeneity, whereby the biology of GBM cells is influenced by their spatial location and their functional interactions with neighboring cells within the tumor microenvironment (TME) 10. Collectively, this multifaceted heterogeneity offers numerous mechanisms for adapting to stress and developing resistance to therapy, contributing to a disease with exceptional resilience. Consequently, gaining a more comprehensive understanding of the heterogeneity of GBM is imperative to enhance patient prognosis.

In the process of transitioning from the initial stage to the adaptive disease stage in GBM, the heterogeneous tumor cells and microenvironment undergo dynamic changes 11. These changes encompass variations in the number of tumor cell subclones and epigenetic alterations in tumor cells. The types and quantities of non-tumor cells comprising the tumor microenvironment (such as pericytes, endothelial cells, glial cells, leukocytes 12 (including dendritic cells 13, 14, neutrophils 15, 16, natural killer (NK) cells 17-19, macrophages 20, 21) and astrocytes 22, 23) also change. Additionally, within the cross-talking between cells, the characteristics of both tumor and non-tumor cells dynamically evolve, directly leading to the tumor's resistance to treatment and malignant progression. For example, tumor-associated macrophages (TAMs), constituting 30-50% of glioma tissue 24, influence surrounding tumor cells by secreting TNFα, activating the NFκB signaling pathway, and inducing their transformation into radioresistant mesenchymal (MES) subtype glioma cells, ultimately impacting patient prognosis 7. As immune cells originating from the myeloid lineage, macrophages infiltrate tumor tissue driven by chemokines such as CSF-1 (Colony-stimulating factor 1) 21, 25, MCP-1 (monocyte chemoattractant protein 1) 26, 27 and SDF-1 (Stromal cell-derived factor 1) 28, 29 secreted by glioma cells, playing roles in anti-tumor (sTAMs, M1; expressing markers like HLA-DR, iNOS, and CD11c ) and pro-tumor (pTAMs, M2; expressing markers like CD163, CD206, and ARG1) functions 15, 30-32. However, the mechanisms regulating how TAMs acquire these different functions remain unclear.

In the tumor microenvironment, different tumor cell subclusters and multiple non-tumor cells contribute to the diversity and complexity of GBM 12, 33, 34. Neglecting this diversity by treating GBM as a uniform entity may overlook critical regulatory mechanisms of distinct tumor cell subclusters. Therefore, investigating the regulatory relationships and networks among these cells is beneficial for effectively inhibiting the malignant progression of the tumor. Recent developments in single-cell sequencing and spatial omics have provided technological support for such research 35, 36. Based on this, we have employed multi-omics and biological validation to study the cell types and molecular mechanisms within the tumor microenvironment that are closely associated with the malignant progression of GBM, with a particular emphasis on the tumor cell types and related mechanisms that play a crucial role in the M2 polarization of TAMs.

## Results

### Enrichment of M2 TAMs is associated with malignant progression of glioma

**Figure 1:**
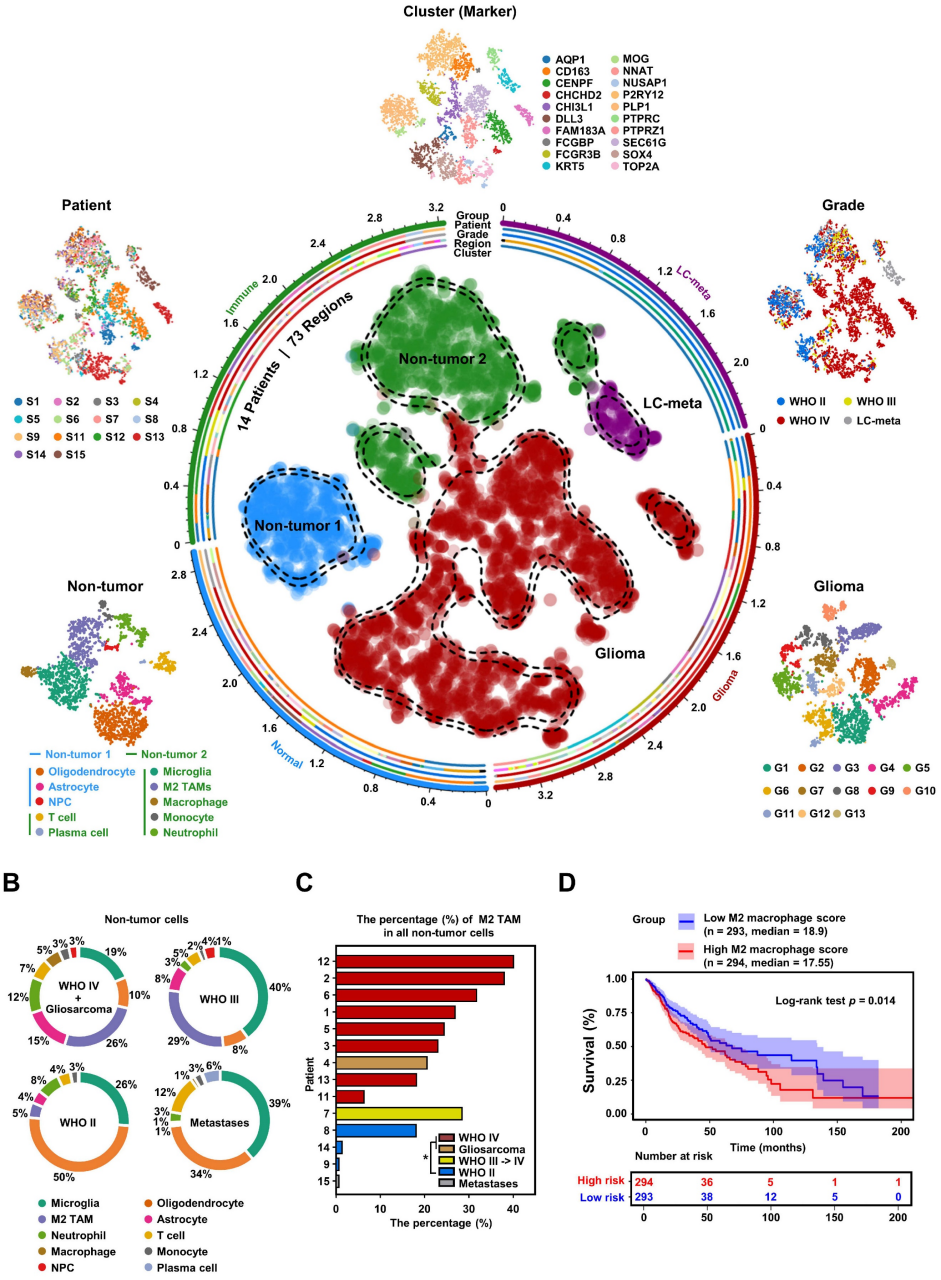
** High-grade gliomas demonstrate significant M2 TAM density. (A)** t-SNE representation of the Gliomap. The corner insets depict the cluster (marker), patient, grade, as well as further subdivisions of non-tumor subclusters and glioma subclusters. The axis outside the circular plot shows the log scale of the total cell number for each cell type (level-3 annotation).** (B)** The pie chart illustrates the distribution of non-tumor cells in different glioma patients (WHO IV, n = 8; Gliosarcoma, n = 1; WHO III->IV, n = 1; WHO II, n = 3; lung cancer metastases, n = 1). **(C)** Histogram shows the percentage (%) of M2 TAMs among all non-tumor cells in 14 glioma patients, colored by different grades. *p < 0.05, two-tailed unpaired t-test.** (D)** The Kaplan-Meier survival curves show that M2 macrophage infiltration scores are associated with malignant progression of glioma in TCGA GBMLGG database. Based on the median value of M2 macrophage score, we divided the patients into high group and low groups. *P* values were determined by log-rank test. Immune infiltration scores are calculated by the CIBERSORT package based on the TCGA GBMLGG expression matrix.

To explore the heterogeneity within the glioma microenvironment, and capture the diversity of cellular states, we needed a tool capable of providing full-length transcript coverage and detecting low-abundance transcripts at the level of individual cells. Therefore, we utilized Smart-seq2 single-cell data from the GEO database to construct a comprehensive glioma map (Gliomap) (**Figure 1A**) 37. The Gliomap was assembled from 14 glioma patients (including 9 with WHO IV grade, 3 with WHO II grade, and 1 with WHO III grade), as well as 1 patient with lung cancer brain metastasis (We included one patient with lung cancer brain metastasis in this study. Subsequent clustering methods were able to distinguish lung cancer metastasis cells from a mixed cell population, demonstrating the reliability of our classification approach) (**Figure S1B**). The map covered 73 sampling regions (**Figure S1A**) and encompassed a total of 6,148 qualified cells. Initially, we categorized all cells into 20 distinct clusters using single-cell RNA sequencing data and quantified Copy Number Variation (CNV) scores for each cluster (**Figure S2A**). Of these, 13 clusters exhibited tumor genomic properties with high CNV scores, while 7 clusters displayed non-tumor genomic properties with low CNV scores (**Figure S2B**). Interestingly, within the tumor clusters, 12 expressed glioma cell marker genes (**Figure S2C** and **Figure S3A**), and one other cluster specifically expressed the lung cancer marker KRT5, indicative of lung cancer metastasis (**Figure S2C**). Among the 7 non-tumor clusters, 5 expressed marker genes for immune cells including M2 TAMs (**Figure S2C**, **S3B**, and **S3C**), and 2 clusters expressed the brain-derived cell marker MOG (**Figure S2C**). Further subclassification of the non-tumor cells revealed that they could be divided into two groups: non-tumor group 1, consisting of brain-derived cells such as oligodendrocytes, astrocytes, and neural progenitor cells; and non-tumor group 2, comprising immune cells such as M2 TAMs, microglia, neutrophils, macrophages, monocytes, and plasma cells (**Figure 1A** and **Figure S4**). Simultaneously, we analyzed the distribution of each non-tumor cluster across different glioma patients. Our analysis revealed that among high-grade glioma patients, M2 TAMs constituted the highest proportion (**Figure 1B**). Furthermore, by applying non-tumor cell-specific gene sets, we simulated the immune cell infiltration proportions in glioma patients within the TCGA GBMLGG database (**Figure S5A-B**). The enrichment of these M2 TAMs demonstrated a dependence on glioma grade, observed consistently across both single-cell and tissue-level data (**Figure 1C**, **Figure S5C-F**), and was associated with poor prognosis for glioma patients (**Figure 1D**). To align with the 2021 WHO classification of tumors of the central nervous system 38, we further examined the IDH1 status and chromosome 1p/19q status of the patients (**Figure S1B, Figure S5G**). These findings from single-cell and tissue-level mRNA sequencing analyses highlight that M2 TAMs within the non-tumor cell population of glioblastoma are significantly linked to the malignancy progression of glioblastoma, illustrating their crucial role in the tumor microenvironment.

### Identification of M2 TAM-related glioma subcluster

**Figure 2:**
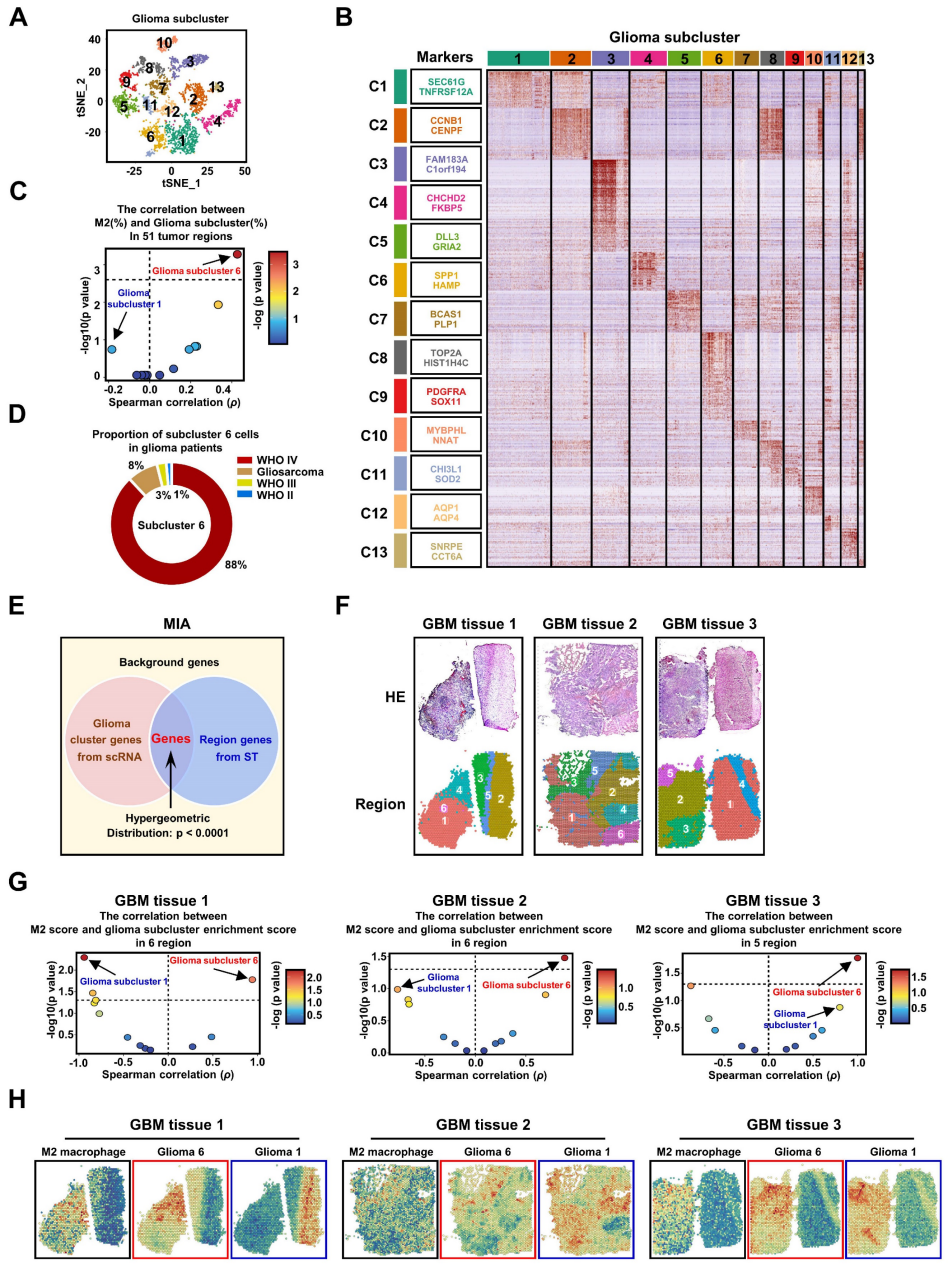
** The GBM subcluster 6 and M2 TAMs exhibit a high correlation in distribution. (A)** All glioma cells were analyzed using t-SNE, and 13 significant cell clusters are color-coded and labeled as indicated. **(B)** The heatmap shows the expression patterns of all marker genes for the 13 glioma subclusters. The boxes (left) contain the top 2 specific markers for each glioma cluster, with the colors indicating the respective glioma subclusters. **(C)** A scatter plot demonstrates the Spearman's rank correlation between the proportions of different glioma subclusters (%) and M2 TAMs (%) across 51 tumor regions, colored by -log10 (*p* value). The x-axis and y-axis represent the correlation coefficient and -log10 (*p* value), respectively. The significance level threshold is set at *p* < 0.05. A correlation coefficient > 0 indicates a positive correlation, while a correlation coefficient < 0 indicates a negative correlation. **(D)** The pie chart displays the proportion of cluster 6 cells in 14 glioma patients. The colors represent different grades of glioma patients. **(E)** The figure is a schematic diagram of the MIA analysis. **(F)** shows spatial transcriptomic analysis of 3 GBM tissues, with the top row showing tissue H&E staining, and the bottom row showing clustering of spatial transcriptomic data. **(G)** The volcano plot displays the spearman correlation between the M2 score and glioma subcluster enrichment score in different regions of the 3 tissues, colored by -log10 (*p* value).** (H)** shows the ssGSEA enrichment score of M2 macrophages, Glioma 6, and Glioma 1 in various regions across the 3 GBM tissues.

To investigate the diversity of tumor cells within gliomas, we subjected all glioma cells to an enhanced clustering process. This analysis resulted in the identification of 13 distinct glioma subclusters, as shown in **Figure 2A**. Each subcluster is characterized by a unique expression profile of marker genes, which are detailed in **Figure 2B** and **Table S2**. Notably, the expression patterns of traditional tumor type markers (**Figure S6A-B**), 3 GBM subtype (**Figure S6C**) and 4 cellular states (**Figure S6D**) typically associated with glioma do not correspond with these newly identified 13 GBM subclusters 5, 37, 39, 40. To explore the connection between different glioma subclusters and M2 TAMs, we computed the correlation between the presence of various glioma subcluster cells and M2 TAMs across 51 tumor regions. Strikingly, our results highlighted that only glioma subcluster 6 exhibited a significant positive correlation with M2 TAMs, while subcluster 1 presented the strongest negative correlation that was not statistically significant. (**Figure 2C**, **Figure S7**). Additionally, the majority of cells in glioma subcluster 6 were found to originate from patients with glioblastoma (GBM, WHO IV grade) (**Figure 2D, Figure S8A**), aligning with our earlier observation that M2 TAMs are predominantly present in high-grade gliomas, particularly GBM. Furthermore, the glioma subcluster 6 gene signature (marker genes) significantly enriched in GBM of larger clinical glioma cohorts (**Figure S8C**), and was associated with the mesenchymal (MES) GBM subtype (**Figure S8D**). Elevated levels of this signature were correlated with poorer prognoses in GBM patients (**Figure S8E-F**). These findings collectively demonstrate that subcluster 6 is prevalent in a majority of GBM patients and is associated with worse outcomes.

Intriguingly, within different regions of the same patient, GBM subcluster 6 exhibited visually observable variations in proportion (**Figure S8A and S8B**), suggesting potential spatial distribution characteristics of subcluster 6 and M2 TAMs within tumor tissue. To further investigate this phenomenon, we analyzed spatial transcriptomic data from three GBM patients. We employed the Multimodal Intersection Analysis (MIA) method, which integrates single-cell sequencing with spatial transcriptomics (**Figure 2E**). This approach was complemented by random partitioning of the spatial transcriptomic data (**Figure 2F, S9A**). Our analysis revealed distinctive distribution patterns of the 13 glioma subclusters and M2 TAMs within the spatially partitioned transcriptomic regions (**Figure S9B**). Specifically, GBM subcluster 6 demonstrated a strong positive correlation with the distribution of M2 TAMs across all examined GBM patient tissues, in contrast to subcluster 1, which exhibited a significant negative correlation in the tissue from GBM patient 1 (**Figure 2G**). Furthermore, the observed co-localization of GBM subcluster 6 and M2 TAMs, as suggested by the imprinting of their signatures in tumor tissues (**Figure 2H**), indicates that the spatial distribution of subcluster 6 may influence the polarization of macrophages toward an M2 phenotype. Currently, some research indicates that M2 TAMs have shown a greater association with the MES subtype 5, 7.

**Figure 3:**
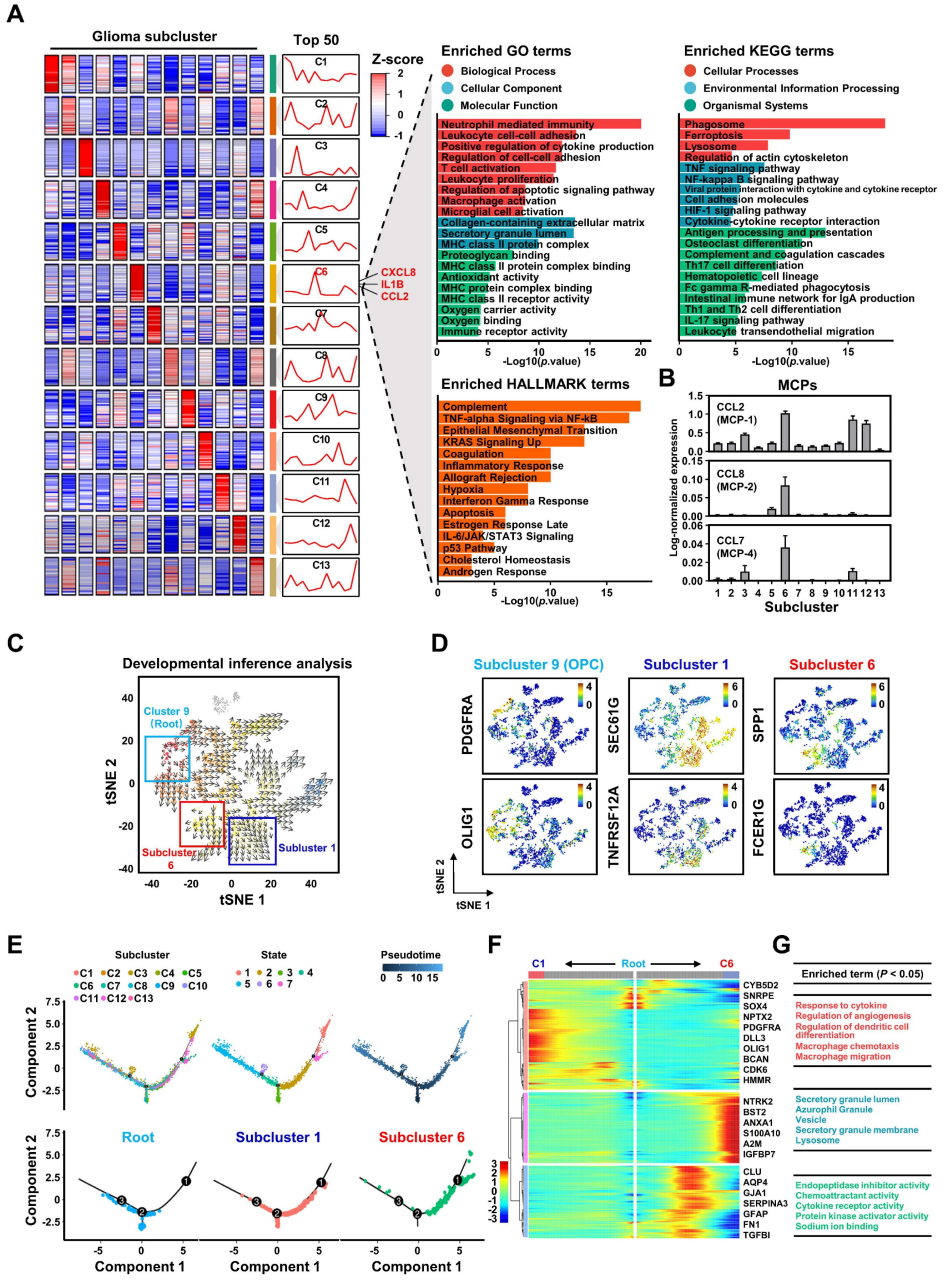
** The biological characteristics of GBM subcluster 6. (A)** Heatmap shows the mean of top50 marker genes of clusters. The line graph represents the differential expression of the mean of these marker genes in all clusters, and on the right side are displayed the ligands associated with M2 macrophage polarization or chemotaxis in subcluster C6. The bar chart represents the functional enrichment of GO (BP, Biological Process; CC, Cellular Component; MF, Molecular Function), KEGG and Hallmark pathways for marker genes in glioma subcluster 6. The x-axis and y-axis represent -log10 (*p* value) and pathways. **(B)** Expression of monocyte chemoattractant protein (MCPs: MCP-1, MCP-2, MCP-4) in different glioma clusters. Data are shown as means ± s.e.m. **(C)** Developmental inference analysis shows the dynamic shift in cell state, with the arrow indicating the direction of cell state transition. **(D)** Feature plot displays represented marker genes for subcluster 9 (PDGFRA, OLIG1), subcluster 1 (SEC61G, TNFRSF12A) and subcluster 6 (SPP1, FCER1G) across all glioma cells. **(E)** The trajectory analysis of all glioma cells is depicted in the first line, with color-coded representation based on glioma clusters, status and pseudotime. The second row displays a trajectory of root, subcluster 1, and subcluster 6. **(F)** Heatmap represents the expression patterns of genes during the developmental process from root to subcluster 1 and subcluster 6. The partial signature genes for each pattern are displayed on the right. **(G)** shows functional enrichment analysis of GO BP (red), GO CC (blue), and GO MF (green) for the gene module of cluster 6.

However, our intriguing finding is that subcluster 6 constitutes only a portion of the cells within the MES subtype (**Figure S10A-B**), implying that studies at the subtype level alone may not be accurate. Therefore, further subdivision of GBM tumor cells is essential for studying the regulatory mechanisms of GBM on M2 macrophage polarization. To delve deeper into the characteristics of GBM subcluster 6, we identified the top 50 highly expressed genes and conducted a functional enrichment analysis. Our results reveal that this subcluster is involved in regulating various biological processes such as TNFα signaling, inflammatory response, hypoxia, apoptosis, and macrophage activation (**Figures 3A** and **S10C**). Notably, the top 50 genes include secreted ligands like CXCL8, IL1B, and CCL2, which are associated with chemotaxis and M2 polarization of macrophages 41-43 (**Figure 3A**). Furthermore, the increased expression of monocyte chemoattractant proteins (MCPs), especially CCL2, suggests a pivotal role for GBM subcluster 6 in macrophage recruitment (**Figure 3B** and **S10D**) 26, 27. Unsupervised developmental inference analysis indicates that various GBM subclusters, including subcluster 6, may originate from GBM subcluster 9, which is characterized by high expression of oligodendrocyte progenitor cell markers such as PDGFRA and OLIG1 (**Figure 3D**). Our analysis shows that GBM subclusters 1 and 6 share closely related developmental trajectories (**Figure 3C**), yet they exhibit distinct gene expression patterns (**Figure 3D**). Additionally, pseudotime analysis suggests that GBM subclusters 1 and 6 share a common evolutionary branch (**Figures 3E** and **S11**), but their genes exhibit opposite expression trajectories during evolution (**Figure 3F**). The genes expressed during the evolution of GBM subcluster 6 are primarily involved in macrophage recruitment and cytokine response **(Figure 3G)**. These evolutionary features may explain the observed distribution and differing overlaps of GBM subclusters 1 and 6 with M2 macrophages in GBM tissues 2 and 3 (**Figure 2H**), highlighting the potential role of subcluster 6 in mechanisms related to macrophage infiltration and activation.

### CEBPB regulates the recruitment and M2 polarization of TAMs as a specific TF-regulon in GBM subcluster 6

**Figure 4:**
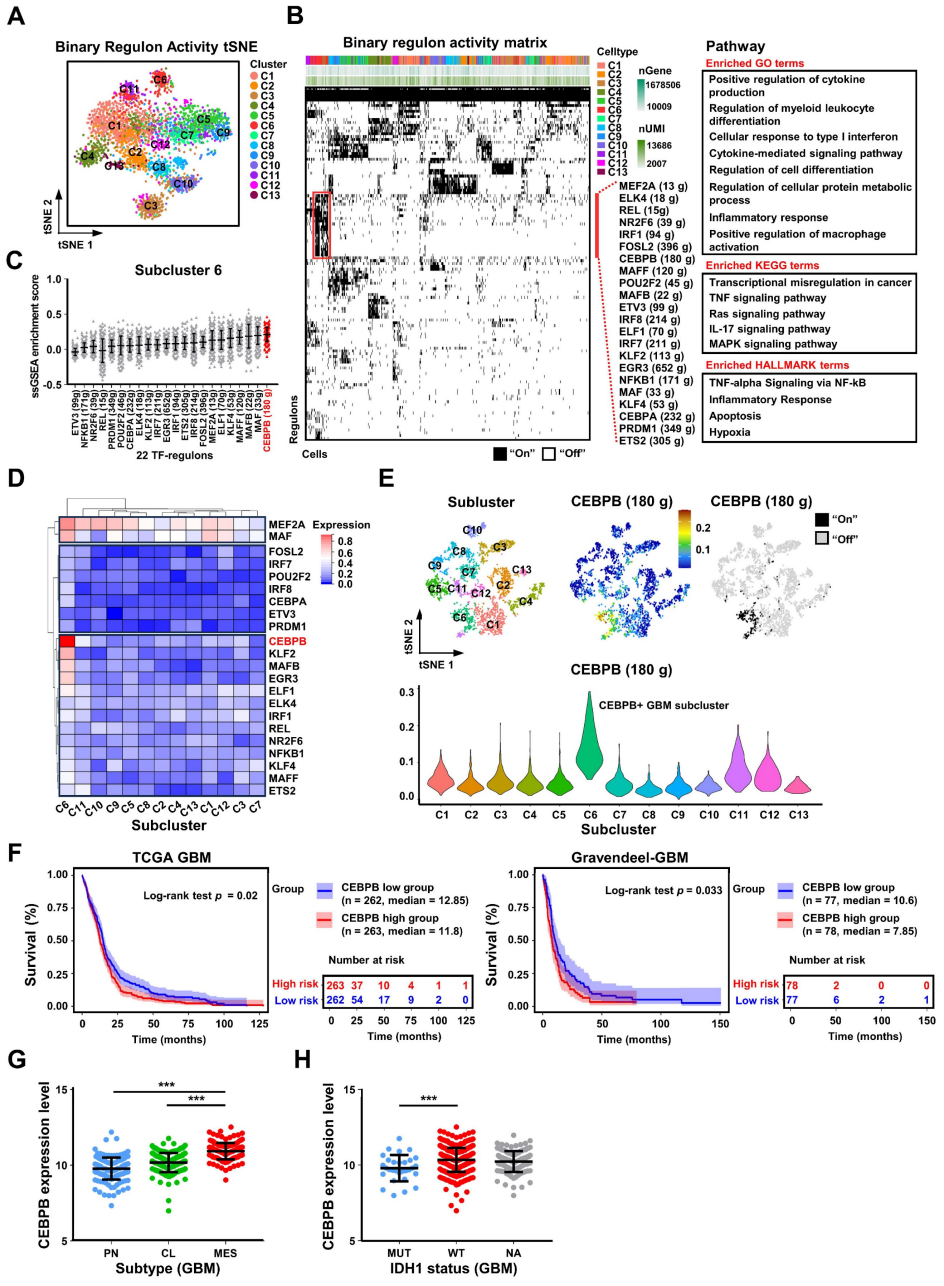
** Single-cell sequencing revealed that CEBPB is a specific TF-regulon of GBM cluster 6. (A)** New t-SNE analysis based on binary regulon activity, analyzed by SCENIC, is color-coded by glioma clusters. **(B)** Binary regulon activity matrix identifies the master TF-regulons in different glioma clusters. On the right, the primary TF-regulons of GBM cluster 6 are listed, along with the number of genes they regulate. Additionally, functional enrichment of GO, KEGG, and HALLMARK pathways associated with these regulons is provided. The pathways shown in the figure have a significance level of *p* < 0.05. **(C)** The scatter plot displays the ssGSEA enrichment scores of 22 TF-regulons in subcluster 6, arranged in ascending order based on their mean values. **(D)** The heatmap displays the relative mRNA expression levels of 22 transcription factors across 13 glioma subclusters. **(E)** The expression distribution of CEBPB-regulons on the original t-SNE coordinates of 13 glioma clusters. The violin plot represents the expression of CEBPB in 13 glioma clusters. **(F)** Kaplan-Meier curves of patient survival stratified by the median of CEBPB expression level from TCGA GBM and Gravendeel-GBM databases. *P* values were determined by log-rank. **(G)** CEBPB expression in subtype (n = 162, PN; n = 198, CL; n = 165, MES) from the TCGA GBM database. Black bars indicate mean ± s.d. ***p < 0.001; one-way ANOVA with Tukey's method for multiple comparisons. **(H)** shows CEBPB expression in GBM patients with IDH1 status (n = 30, IDH1 mutation (MUT); n = 372, IDH1 wild type (WT); n = 123, unknown (NA)) in the TCGA GBM database. Data are represented as means ± s.d. ***p < 0.001; two-tailed unpaired t-test.

The destiny and function of a cell are determined by coordinated gene networks. Transcription factor regulons (TF-regulons), which serve as composite analytical units encompassing transcription factors and their direct target genes, can be employed to analyze the master regulators within gene networks governing cellular biological processes 44. Using SCENIC 45, 46, we computed transcription factor regulons (TF-regulons) from single-cell sequencing data, effectively distinguishing the 13 glioma subclusters through binary regulon activity (**Figure 4A-B**). We identified 22 distinct TF-regulons exclusive to GBM subcluster 6, associated with specific biological functions including macrophage activation (**Figure 4B**, **Figure S12A**). Remarkably, among these 22 TF-regulons, the regulon of CCAAT/Enhancer-Binding Protein Beta (CEBPB) exhibited the highest coverage in GBM subcluster 6 (**Figures 4C**, **S12B**, and **S12C**). Additionally, CEBPB mRNA expression was the highest in this subcluster compared to other transcription factors (**Figure 4D**). Subsequently, we mapped the transcriptional regulatory pattern of the CEBPB-regulon onto the single-cell RNA-seq data, confirming its specific expression in GBM subcluster 6 (**Figure 4E**). To assess the potential clinical implications of CEBPB, we investigated the connection between CEBPB expression and outcomes for GBM patients. Elevated CEBPB expression was associated with an unfavorable prognosis in GBM patients across various databases, including TCGA GBM and Gravendeel (only GBM patients) databases (**Figure 4F**). Furthermore, CEBPB expression displayed a positive correlation with the malignant mesenchymal subtype (MES), as well as the malignant cases of IDH1 wild type and within the TCGA GBM database (**Figure 4G-H**). In summary, we propose that CEBPB functions as a specific TF-regulon for GBM subcluster 6, governing its transcriptional network and potentially participating in initiating the recruitment and activation of TAMs.

**Figure 5:**
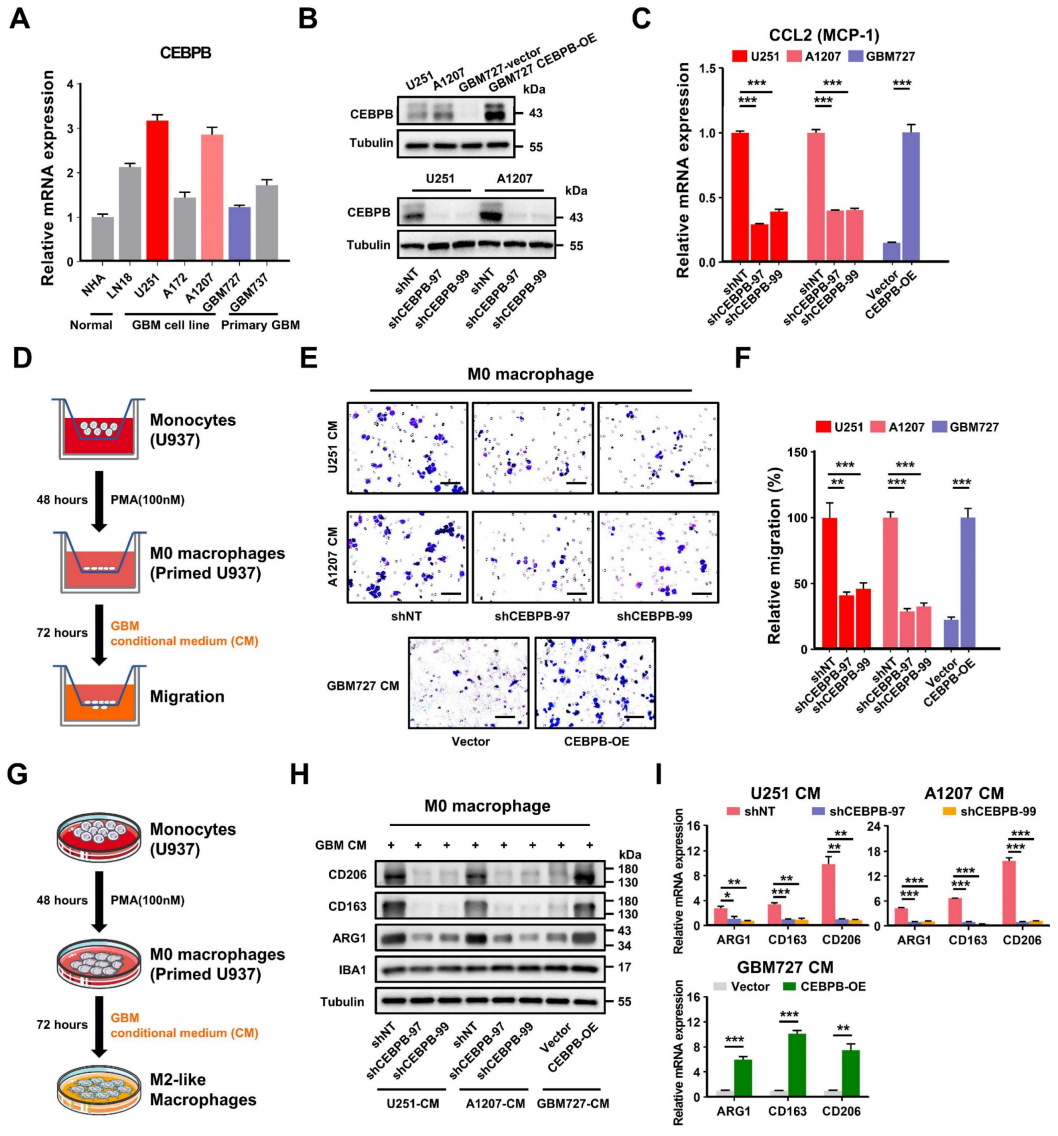
** CEBPB can recruit TAMs and polarize them towards the M2 phenotype in vitro. (A)** The bar graph shows the relative mRNA expression levels of CEBPB in normal tissues, GBM cell lines and primary GBM by qPCR. Data are represented as means ± s.e.m. n = 3 independent experiments. **(B)** Immunoblot analysis of CEBPB expression in GBM cells (U251, A1207, GBM727-Vector, GBM727-CEBPB-overexpression (CEBPB-OE)) (top) and GBM cells (U251 and A1207) transduced with non-targeting shRNA (shNT) or CEBPB shRNA (shCEBPB) through lentiviral infection (bottom). **(C)** Relative mRNA expression of CCL2 (MCP-1) expression in GBM cells (U251 and A1207) transduced with non-targeting shRNA (shNT) or CEBPB shRNA (shCEBPB) through lentiviral infection and GBM727- Vector, GBM727-CEBPB-OE. Data are represented as means ± s.e.m. n = 3 independent experiments. ***p < 0.001. Statistical significance was determined by one-way ANOVA analysis. **(D)** A schematic diagram for migration experiment of M0 macrophages (U937-derived) in vitro. **(E)** Representative images show M0 macrophages (U937 differentiated into macrophages after treatment with 100 nM PMA) that migrated towards GBM conditional media. Scale bar, 100 µm. **(F)** Graphical analysis of **(E)** displays a significant reduction of macrophages that migrated towards GBM conditioned media expressing shCEBPB. **p < 0.01, ***p < 0.001 (n = 5 fields); mean ± s.e.m; two-tailed unpaired t-test. **(G)** A schematic diagram for M2 polarization of macrophages (U937-derived) in vitro. **(H)** Western blotting and **(I)** qPCR were used to detect the expression of M2 markers (CD206, CD163 and ARG1) and the total macrophage marker IBA1 in M0 macrophage (U937 differentiated into macrophages after treatment with 100nM PMA) treated with GBM conditional media for 72 h. α-tubulin was blotted as the loading control. Data are represented as means ± s.e.m. n = 3 independent experiments. **p < 0.01, ***p < 0.001. Statistical significance was determined by one-way ANOVA analysis.

However, the culturing conditions for tumor cells in vitro differ markedly from the tumor microenvironment in vivo, which may hinder the precise emulation of specific cellular clusters within the tumor. Nonetheless, the results of high-throughput sequencing suggest a positive correlation between the high expression of CEBPB in GBM subcluster 6 and M2 TAMs. Therefore, we assessed the CEBPB expression levels in 1 normal cell line, 4 GBM cell lines, and 2 primary GBM cells. By comparing the expression levels of CEBPB in these samples, we selected U251 and A1207, which have high CEBPB expression, as positive models, while designating GBM727, which exhibits low CEBPB expression, as a negative model (**Figure 5A**). Subsequently, we knocked down CEBPB in U251 and A1207 cells and overexpressed CEBPB in GBM727 cells. (**Figure 5B**, **Figure S13A**).

Depletion of CEBPB in these two GBM cell lines not only significantly decelerated the growth of GBM cells but also markedly decreased the expression level of monocyte chemoattractant protein-1 (CCL2) (**Figure 5C**, **Figure S13B-C**), a crucial factor in mediating the chemotactic migration of macrophages. Conversely, upon overexpression of CEBPB in GBM727, the expression level of CCL2 was also significantly increased (**Figure 5C**). Subsequent Transwell cell migration assays demonstrated that conditioned medium (CM) from GBM cells overexpressing CEBPB significantly enhanced the migration of M0 macrophages (PMA-primed U937 cells). (**Figure 5D-F**). Interestingly, following prolonged exposure (3 days) of M0 macrophages to conditioned media from GBM cells overexpressing CEBPB, the expression of M2-like markers CD206, CD163, and ARG1 sharply increased. In contrast, this trend was notably diminished upon exposure to conditioned media from CEBPB-depleted GBM cells, while there was no change in the expression of M1-like markers (iNOS, TNFα, and CD80) (**Figure 5G-I, Figure S13D**). These results suggest that the genes regulated by CEBPB might encompass factors involved in M2 polarization of macrophages.

**Figure 6:**
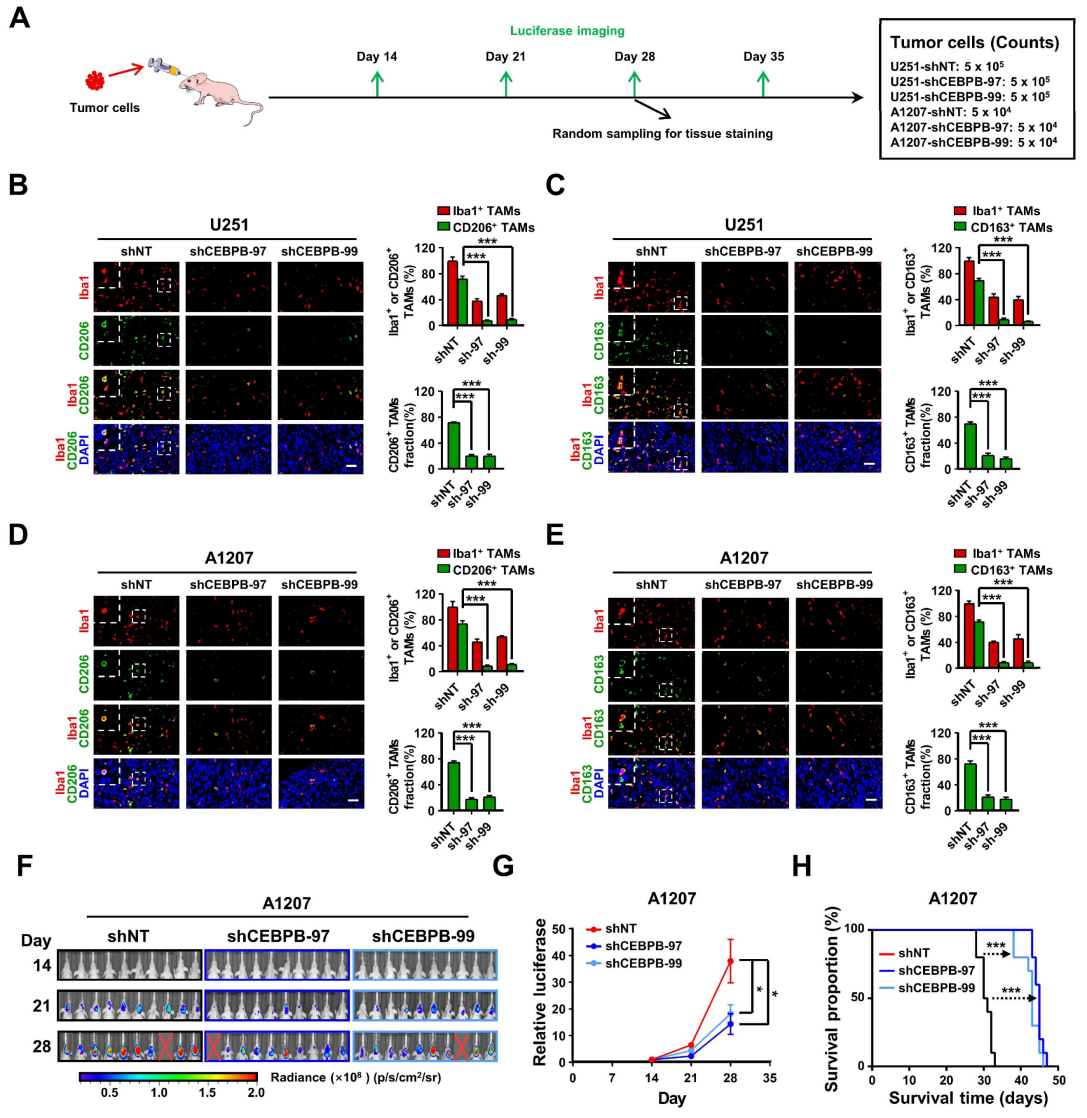
** CEBPB triggers M2 polarization of TAMs to promote malignancy growth in vivo. (A)** Experimental design to assess CEBPB triggers M2 polarization of TAMs in vivo. **(B)**-**(E)** Immunofluorescent staining of the M2 TAM Marker (CD206 and CD163) (green) and the pan-macrophage marker Iba1 (red) in GBM xenografts derived from U251 and A1207 expressing shNT control or shCEBPB. Boxed areas are further magnified. Scale Bar, 40 μM. Histogram show the quantitation of M2 TAM density and the fraction of M2 TAMs in xenografts derived from U251 and A1207 expressing shNT or shCEBPB. N = 5 (shNT, shCEBPB-97 or shCEBPB-99) biological independent tumor samples. The M2 TAM fraction was determined by the percentage of M2 TAMs within TAMs in shNT or shCEBPB xenografts, respectively. Data are represented as means ± s.e.m. ***p <0.001, two-tailed unpaired t-test. **(F)**-**(H)** Left, representative images on day 14, 21, 28 post transplantation are shown; bioluminescence is measured in p/s/cm^2^/sr. Middle, quantification of relative luciferase signals during 28 days. A1207: shNT (n = 9), shCEBPB-97 (n = 9), shCEBPB-99 (n = 9); Data are represented as means ± s.e.m. *p < 0.05, one-way ANOVA with Tukey's method for multiple comparisons. Right, Kaplan-Meier survival curves of mice bearing A1207-derived xenografts expressing shNT or shCEBPB. ***p < 0.001, log-rank test. A1207: shNT (n = 10), shCEBPB-97 (n = 10), shCEBPB-99 (n = 10).

To validate our in vitro findings using animal models, we established orthotopic xenograft models utilizing U251 and A1207 cells with or without CEBPB depletion (**Figure 6A**). After 28 days post-transplantation, we randomly collected mouse brain tissues for the evaluation of Iba1 (the total macrophage marker) 31, 32, 47, CD206 and CD163 positive TAMs through immunofluorescence staining. Consistent with our in vitro results, the depletion of CEBPB in transplanted glioma cells not only significantly reduced the overall TAMs (Iba1 positive) content but also markedly decreased the content of CD206 or CD163 positive M2 TAMs (**Figure 6B-E**). These findings suggest that CEBPB is also implicated in TAMs recruitment and M2 polarization in vivo. The subsequent in vivo experiment demonstrated that the depletion of CEBPB in transplanted glioma cells visually reduced the growth of the tumors and extended the survival time of the mice bearing glioma cells (**Figure 6F-H, Figure S13E-G**). The results from our in vitro and in vivo experiments involving CEBPB demonstrate that the high expression of CEBPB in GBM subcluster 6 not only contributes to glioma cell growth but also enhances glioma malignancy by influencing the recruitment and M2 polarization of TAMs in the tumor microenvironment.

### CEBPB transcriptionally targets SPP1 in CEBPB^+^ GBM cluster for inducing M2 polarization of TAMs through Integrin αvβ1-Akt signaling

**Figure 7:**
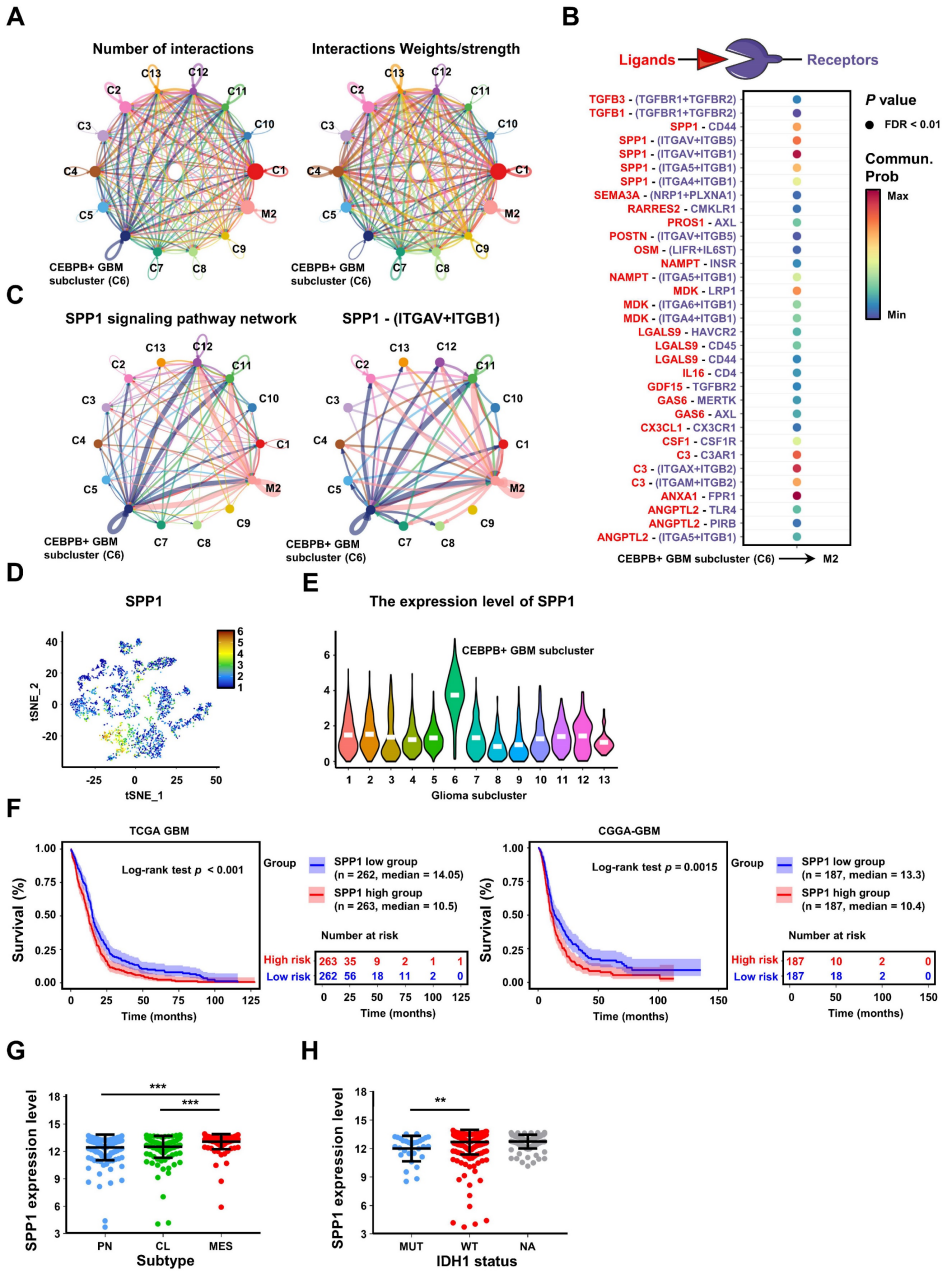
** Intercellular communications show that SPP1 secreted by CEBPB^+^ GBM subcluster may regulate M2 TAMs. (A)** A summary of cell communication between M2 TAMs and 13 glioma clusters. The Number of interactions indicates the quantity of distinct signaling pathways between each pair of clusters. The Interactions Weights/strength reflects the intensity or significance of these interactions, which might be calculated based on the expression levels of signaling molecules or other metrics. **(B)** Bubble plot shows the potential ligand-receptor interactions between CEBPB^+^ GBM subcluster and M2 TAMs. The dot color and size represent the calculated communication probability and *p* values. *P* values are computed from one-sided permutation test. **(C)** The inferred SPP1 signaling pathway network and SPP1 - (ITGAV+ITGB1) interaction network. Circle sizes are proportional to the number of cells in each cell cluster and edge width represents the communication probability. **(D)** The expression distribution of SPP1 on t-SNE coordinates and **(E)** their expression in various glioma clusters. **(F)** Kaplan-Meier curves of patient survival stratified by the median of SPP1 expression level from TCGA GBM and CGGA-GBM databases. *P* values were determined by log-rank. **(G)** SPP1 expression in subtype (n = 162, PN; n = 198, CL; n = 165, MES) from the TCGA GBM database. Black bars indicate mean ± s.d. ***p < 0.001; one-way ANOVA with Tukey's method for multiple comparisons. **(H)** shows SPP1 expression in GBM patients with IDH1 status (n = 30, MUT; n = 372, WT; n = 123, NA) in the TCGA GBM database. Data are represented as means ± s.d. **p < 0.01; two-tailed unpaired t-test.

To further explore the potential regulatory mechanism of CEBPB^+^ GBM subcluster in inducing M2 polarization of TAMs, we employed CellChat 48 to predict the ligand-receptor interactions between the 13 glioma subclusters and M2 TAMs (**Figure 7A, Figure S14A**), and identified 33 potential ligand-receptor interaction pairs between CEBPB^+^ GBM subcluster and M2 TAMs, with the pairs SPP1-Integrin αvβ1 and ANXA1-FPR1 exhibiting the strongest interactions (**Figure 7B-C**, **Figure S14B,D**). We also found that SPP1 can act on M2 TAMs in an autocrine manner, which is consistent with existing studies on SPP1^+^ TAMs (**Figure 7C, Figure S14C**) 49. These pairs also ranked as the top two interaction between CEBPB^+^ GBM subcluster and all types of macrophages (**Table S3**). However, in the single-cell level expression patterns of these factors, we observed that only SPP1 was specifically expressed in CEBPB^+^ GBM subcluster (**Figure 7D-E**), whereas the ANXA1 did not exhibit this cell-type-specific expression pattern (**Figure S14E**). Additionally, we know that glioma cells secrete multiple ligands that act on macrophages to promote their M2 polarization. Interestingly, we found that most of these ligands (e.g., CSF1, CXCL8, POSTN) 21, 32, 43 are broadly expressed in different glioma subclusters, whereas SPP1 is specifically expressed in the CEBPB^+^ GBM subcluster (**Figure S14F**). Moreover, the high expression of SPP1 is closely associated with the GBM subtypes, IDH1 status and adverse patient prognosis (**Figure 7F-H**). These results, based on in silico simulations, suggest that the SPP1-Integrin αvβ1 pair plays a crucial intermediary role in the interaction between CEBPB^+^ GBM subcluster and M2 TAMs for glioma malignant progress.

**Figure 8:**
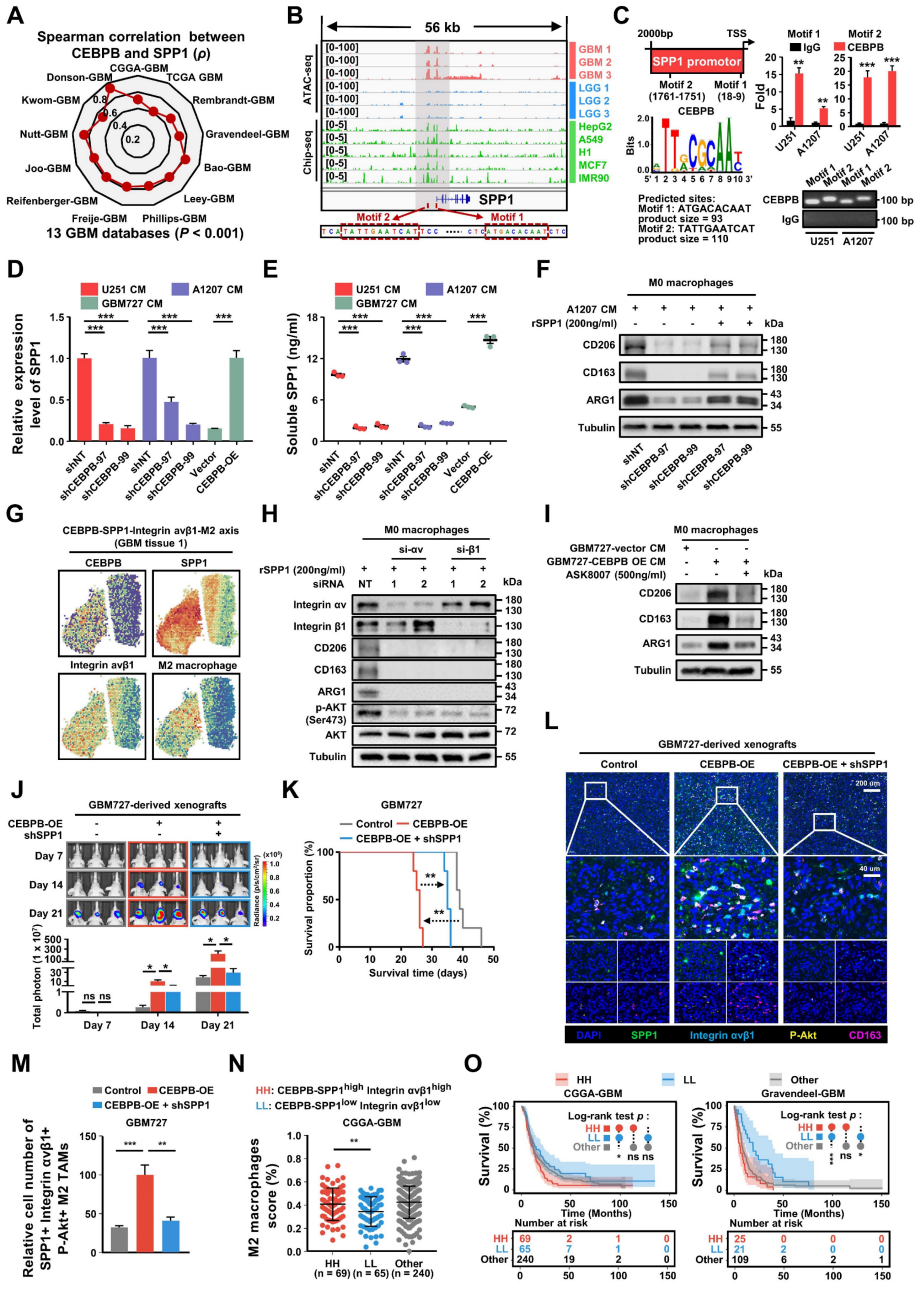
** GBM cluster 6 induce M2 polarization of TAMs through SPP1-Integrin αvβ1-Akt axis. (A)** The radar chart shows the Spearman's rank correlation between CEBPB and SPP1 expression in 13 GBM databases. **(B)** IGV visualization shows ATAC-seq (Data range: 0-100) of different grade gliomas (GBM, red; LGG, blue) and ChIP-seq (Data range: 0-5) of CEBPB in different cell lines (green) at the SPP1 promoter region. The red box below indicates the predicted binding site of CEBPB motif in the promoter region of SPP1. **(C)** Predicted CEBPB motif in the promoter region of SPP1. CUT&RUN-qPCR and gel electrophoresis show transcription factor CEBPB binds directly to promoter regions of SPP1. Cross-linked chromatin was prepared from U251 and A1207. *P* values were calculated using the 2-tailed 2-sample t test. Data are shown as means ± s.e.m. n = 3 independent experiments. **p < 0.01, ***p < 0.001. **(D)** qPCR shows the mRNA expression level of SPP1 in U251(shNT, shCEBPB), A1207 (shNT, shCEBPB) and GBM727 (Vector, CEBPB-OE). Data are shown as means ± s.e.m. n = 3 independent experiments. Statistical significance was determined by one-way ANOVA analysis.** (E)** Analysis of the changes in SPP1 production in U251(shNT, shCEBPB), A1207 (shNT, shCEBPB) and GBM727 (Vector, CEBPB-OE) at 48 h using ELISA (cells were seeded at 0.5 × 10^6^/ml as a starting culture density). *P* values were calculated using the 2-tailed 2-sample t test. Data indicate mean ± s.e.m and are representative of 3 independent experiments. ***p < 0.001. **(F)** Immunoblot analysis of M2 macrophages markers (CD206, CD163 and ARG1) in M0 macrophages (primed-U937 cells) treated with A1207 GBM CM and 200ng/ml rSPP1 protein for 72 h. α-tubulin were blotted as the loading control.** (G)** The spatial transcriptomics data demonstrated the co-localization of the CEBPB-SPP1-Integrin αvβ1-M2 axis.** (H)** Immunoblot analysis of M2 macrophages marker and Akt phosphorylation (Ser473) in M0 macrophages (primed-U937 cells) expressing si-Integrin αv or si-Integrin β1. These cells were then treated with a concentration of 200 ng/mL of the recombinant SPP1 (rSPP1) protein for 72 h. **(I)** Immunoblot analysis of M2 macrophages marker in M0 macrophages (primed-U937 cells) treated with GBM CM (GBM737-NT CM and GBM737-CEBPB-OE CM) and ASK8007. **(J)** Top, representative images on day 7, 14, 21 post transplantation are shown; bioluminescence is measured in p/s/cm2/sr. Bottom, quantification of relative luciferase signals during 21 days. GBM727: Control (n = 3), CEBPB-OE (n = 3), CEBPB-OE + shSPP1 (n = 3); Data are represented as means ± s.e.m. *p < 0.05; ns, *p* > 0.05, one-way ANOVA with Tukey's method for multiple comparisons.** (K)** Kaplan-Meier survival curves of mice bearing GBM727-derived xenografts (Control, CEBPB-OE, CEBPB-OE + shSPP1). **p < 0.01, log-rank test. GBM727: Control (n = 5), CEBPB-OE (n = 5), CEBPB-OE + shSPP1 (n = 5). Representative images from multiplex immunofluorescence **(L)** and statistical data **(M)** show the relative cell number of SPP1^+^ Integrin avβ1^+^ CD163^+^ P-Akt^+^ M2 TAMs in GBM727 (Control, n = 5; CEBPB-OE, n = 5; CEBPB-OE + shSPP1, n = 5). Boxed areas are further magnified. Scale Bar, 200uM or 40μM. *P* values were calculated using the 2-tailed 2-sample t test. Data are shown as means ± sem. **p < 0.01, ***p < 0.001.** (N)** The differences in the infiltration score (%) of M2 macrophages among the different groups (HH: CEBPB-SPP1^high^ Integrin αvβ1^high^, LL: CEBPB-SPP1^low^ Integrin αvβ1^low^, Other) in the CGGA-GBM database. *P* values were calculated using the 2-tailed 2-sample t test. Data are shown as means ± sd. **p < 0.01. **(O)** Kaplan-Meier survival analysis of 3 defined groups (CEBPB-SPP1^high^ Integrin αvβ1^high^, CEBPB-SPP1^low^ Integrin αvβ1^low^, Other) in the CGGA-GBM, and Gravendeel-GBM databases. *P* values were determined by log-rank. *p < 0.05, ***p < 0.001, ns: *p* > 0.05.

To assess the relationship between the major transcription factor CEBPB and SPP1 in CEBPB^+^ GBM subcluster, we initially analyzed the correlation between the expressions of CEBPB and SPP1 in 13 different GBM transcriptome databases. The results show that the expression of CEBPB and SPP1 is highly positively correlated across all GBM databases. (*p* < 0.001) (**Figure 8A**). Furthermore, when comparing chromatin accessibility analysis between GBM and low-grade glioma (LGG), it was discovered that the SPP1 promoter region contains two GBM-specific motifs, which are consistent with the binding sites of CEBPB in other cell types (**Figure 8B**). Our CUT&RUN experiments further confirmed that CEBPB in GBM cells specifically binds to two motifs of SPP1 (**Figure 8C**), and the expression level of CEBPB directly influences SPP1 expression at both mRNA and protein levels (**Figure 8D-E**, **Figure S15A**). Additionally, recombinant SPP1 protein can directly induce M2 polarization of M0 macrophages (**Figure S15B**). Following the knockdown of CEBPB, treatment with recombinant SPP1 successfully restored M2 polarization in M0 macrophages (**Figure 8F**).

Consistent with our previous findings on intercellular communication, we observed co-localization of CEBPB, SPP1, Integrin αvβ1, and M2 macrophages in their spatial distribution (**Figure 8G**). Based on these observations, we hypothesize that SPP1 may influence M2 polarization of macrophages by binding to Integrin αvβ1. After blocking Integrin αv or β1 with siRNA, we found that M2 polarization of macrophages was inhibited, and downstream AKT activation was also suppressed (**Figure 8H**). Next, we treated GBM727-CEBPB-OE conditioned medium (CM) with the SPP1 inhibitor ASK8007 and found that inhibiting SPP1 could reverse the M2 polarization of macrophages induced by the overexpression of CEBPB (**Figure 8I**, **Figure S15C**). However, since ASK8007 cannot cross the blood-brain barrier, we used shRNA to knock down SPP1 in GBM727-CEBPB-OE cells. We discovered that inhibiting SPP1 reversed the tumor progression and extended survival times caused by the overexpression of CEBPB in vivo (**Figure 8J-K**), which was associated to change the number of SPP1/Integrin αvβ1/phosphorylated-Akt-positive M2 TAMs in the xenograft tumors (**Figure 8L-M, Figure S16**). Clinically, simultaneous high expression of CEBPB, SPP1, and Integrin αvβ1 not only leads to a high enrichment of M2 TAMs in GBM tissues but also significantly shortens the lifespan of GBM patients (**Figure 8N-O**, **Figure S15D-E**). These results indicate that CEBPB, as the major transcriptional regulator in CEBPB^+^ GBM subcluster, influences M2 polarization of TAMs by secreting SPP1 that targets the Integrin αvβ1 receptors on TAMs, thereby activating the downstream AKT signaling pathway, and this molecular mechanism directly contributes to a poor prognosis in GBM patients.

## Discussion

Comprehending the intricate interactions among diverse cell clusters within the microenvironment of GBM plays a pivotal role in advancing our understanding of the dynamics of heterogeneous tumor progression and in devising corresponding therapeutic strategies 50. This article primarily delves into the mechanisms behind the formation of M2 TAMs, which are closely associated with the malignant progression of glioblastoma. It identifies CEBPB as a major transcriptional factor in GBM subcluster 6 and demonstrates how this subcluster orchestrates the recruitment and polarization of macrophages through MCP1 and SPP1, ultimately leading to their transformation into M2 TAMs. This discovery not only enriches our comprehension of M2 TAM formation but also offers novel insights into controlling the malignant progression of GBM.

Accumulated evidence suggests that M2 TAMs play a significant role in promoting the growth, tumor angiogenesis, immune evasion, and treatment resistance of GBM 7, 51. Although a few articles have suggested that GBM cells secrete Periostin (POSTN) and Inducible Signaling Pathway Protein 1 (WISP1) to recruit and polarize M2 macrophages 31, 32. However, GBM is not a single tumor composed of cells with identical genetic and epigenetic characteristics; instead, it is a highly heterogeneous tumor comprising tumor cells with different genetic mutations and expression profiles 52, 53. In fact, through single-cell sequencing, we have identified 13 different subclusters of glioma cells within GBM, with only GBM subcluster 6 exhibiting a strong correlation with M2 TAMs. This more refined and specific classification approach, in contrast to current methods such as Suva or Verhaak classifications, enables us to delve more deeply into the interactions between various cell types within GBM 5, 39.

As a specific transcriptional regulon within GBM subcluster 6, CEBPB plays a crucial role in determining the tumor characteristics of this subgroup. CEBPB, a transcription factor belonging to the C/EBP family, directly modulates the transcription of genes involved in immune and inflammatory responses, particularly in immune cells such as macrophages 54. It is also engaged in diverse cellular processes, encompassing cell proliferation, differentiation, apoptosis, and aging 55, 56. In the context of GBM, CEBPB not only governs the proliferation, migration, and invasion of glioma cells 57, but it is also closely linked with the MES subtype of GBM, correlating with unfavorable clinical outcomes 58. The MES subtype is characterized by the significant infiltration of M2 tumor-associated macrophages (TAMs) and hypoxia, leading to the reconstruction of a distinctive immune-resistant microenvironment 7. Moreover, the functional enrichment analysis of genes associated with GBM subcluster 6 suggests a potential association with MES subtype characteristics. This hints at the possibility that GBM subcluster 6 might be a component of the MES subtype in GBM. However, the precise regulatory mechanisms through which the MES subtype reshapes the immune microenvironment of GBM remain incompletely understood. Our study illuminates the role of CEBPB in GBM subcluster 6, demonstrating its ability to recruit and polarize macrophages into the M2 phenotype by regulating the secretion of CCL2 and SPP1 by tumor cells. Consequently, our research not only enhances our understanding of CEBPB's impact on tumor cells but also sheds light on its contribution to modifying the tumor microenvironment, thereby fostering the overall malignant progression of tumors during the carcinogenic process.

While it has been reported that SPP1 plays an important role in inducing and maintaining M2 macrophage polarization 59, 60, the specific receptors and signaling pathways involved remain unclear. The arginine-glycine-aspartate (RGD) domain within SPP1 has the potential to bind to integrins 61. We have not only demonstrated the transcriptional regulation of SPP1 by CEBPB but have also confirmed that the SPP1-Integrin αvβ1-AKT signaling pathway is applicable to M2 polarization of TAMs in GBM.

Furthermore, we have discovered that GBM subcluster 6 and M2 TAMs may mutually influence each other through SPP1's autocrine mechanism (as shown in **Figure 7C**). This suggests that SPP1's autocrine secretion not only participates in the maintenance of M2 macrophages to form SPP1^+^ TAMs but may also promote the development of GBM subcluster 6 through the Integrin αvβ1-AKT signaling pathway. RGD, as a competitive inhibitor of SPP1, may be a potential candidate for inhibiting GBM subcluster 6 and M2 TAM polarization.

In conclusion, our study has provided new insights into the regulation of M2 TAM formation by specific tumor cell subclusters. This mechanism-oriented research, grounded in the diversity and interactions among cells within the tumor, not only advances our understanding of the progression of tumor malignancy but also paves the way for enhancing current, overly simplistic GBM treatment strategies.

## Materials and Methods

### Data accessibility

The scRNA-seq data of glioma samples (GSE117891) were obtained from Gene Expression Omnibus (GEO, http://www.ncbi.nlm.nih.gov/geo/) database. The bulk RNA-seq expression data and phenotype information of glioma were obtained from GlioVis (http://gliovis.bioinfo.cnio.es/). The spatial transcriptomics (ST) data for glioma were obtained using the 10X genomics datasets (https://www.10xgenomics.com/cn) and GSE235672. The ATAC-seq data for patients with different grades of glioma were obtained from the TCGA database (https://gdc.cancer.gov/about-data/publications/ATACseq-AWG). The ChIP-seq data for the transcription factor CEBPB was obtained from the ENCODE project (https://www.encodeproject.org/).The immunohistochemistry data for CEBPB in glioma patients was obtained from The Human Protein Atlas (https://www.proteinatlas.org/).

### Bioinformatics analysis

All bioinformatics analyses can be found in the **Supplementary Information**, including single-cell RNA sequencing data analysis, spatial transcriptomics data analysis, and other analyses.

### Cell lines and culture condition

All cells used in the study were validated by short tandem repeat (STR) profiling. All cells were cultured in a humidified incubator at 37°C with 5% CO2 and atmospheric oxygen. The ATCC cells (U251, A1207 and 293FT) were cultured in Dulbecco's Modified Eagle Medium (DMEM, Gibco, 11995500) supplemented with 10% fetal bovine serum (FBS, Mei5bio, MF443) and 1% penicillin-streptomycin solution (Bioss, C7072). Human U937 cells were maintained in RPMI 1640 medium (Gibco, 11875500) with 10% FBS and 1% penicillin-streptomycin solution. Human primary GBM cells (GBM727 and GBM737) are derived from human primary GBM specimens. These GBM samples were collected at the Department of Neuro-Oncology and Neurosurgery, Tianjin Medical University Cancer Institute and Hospital in accordance with the Institutional Review Board-approved protocol. The primary GBM cells were recovered in Neurobasal-A medium (Gibco) with B27 supplement (Gibco), 10 ng/ml EGF (Gold Biotech), 10 ng/ml bFGF (R&D), 1 mM sodium pyruvate (Gibco), and 2 mM L-glutamine (Gibco).

### Realtime-qPCR analysis

To confirm the mRNA expression levels of the gene, we used RT-qPCR analysis to determine the gene expression. qPCR primers were designed to span an intron of each target gene. The total mRNA was extracted and purified using a cellular RNA extraction kit (SparkJade, AC0205-B). mRNA (500 ng) was reverse transcribed into cDNA with UEIris RT mix with DNase kit (Us EVERBRIGHT, R2020) on a T20 thermal cycler (LongGene). RT-qPCR assays were performed with Universal SYBR Green qPCR Supermix (Us EVERBRIGHT, S2024) on a 7900 thermal cycler (Applied Biosystems). Three-step amplification was performed (95°C 30 s, 60°C 10 s, and 72°C 30 s) for 32 cycles. For data analysis, expression values were normalized to 18S and RT-qPCR repeated three times. Gene-specific primers as follows: 18S forward 5′-TGCATGGCCGTTCTTAGTTG-3′ and reverse 5′-AGTTAGCATGCCAGAGTCTC-3′, CEBPB forward 5′-AGAAGACCGTGGACAAGCACAG-3′ and reverse 5′-CTCCAGGACCTTGTGCTGCGT-3′; SPP1 forward 5′-CGAGGTGATAGTGTGGTTTATGG-3′ and reverse 5′-GCACCATTCAACTCCTCGCTTTC-3′; MRC1 (CD206) forward 5′-GCCAAATGACGAATTGTGGA-3′ and reverse 5′-CACGAAGCCATTTGGTAAACG-3′; CD163 forward 5′-TTTGTCAACTTGAGTCCCTTCAC-3′ and reverse 5′-TCCCGCTACACTTGTTTTCAC-3′; ARG1 forward 5′-ACTTAAAGAACAAGAGTGTGATGTG-3′ and reverse 5′-CATGGCCAGAGATGCTTCCA-3′. CCL2 forward 5′-AGAATCACCAGCAGCAAGTGTCC-3′ and reverse 5′-TCCTGAACCCACTTCTGCTTGG-3′; iNOS forward 5′-GTTCTCAAGGCACAGGTCTC-3′ and reverse 5′-GCAGGTCACTTATGTCACTTATC-3′; TNFα forward 5′-CCTCTCTCTAATCAGCCCTCTG-3′ and reverse 5′-GAGGACCTGGGAGTAGATGAG-3′; CD80 forward 5′-CTCTTGGTGCTGGCTGGTCTTT-3′ and reverse 5′-GCCAGTAGATGCGAGTTTGTGC-3′.

### Immunoblot analysis

Cells were collected and lysed in RIPA buffer (Thermo Scientific) containing phosSTOP phosphatase inhibitor cocktail (Roche) and protease inhibitor cocktail (Sigma) and separated by SDS-PAGE (NuPAGE Bis-Tris gel, Invitrogen) and transferred to NC membranes (Millipore). After blocking with 5% (wt/vol) non-fat milk in TBS + Tween-20 (0.5% vol/vol), the membranes were probed with primary antibodies against CEBPB (1:1,000, Santa Cruz, sc-7962), CD163 (1:1,000, Abcam, ab182422), CD206 (1:1,000, Abcam, ab64693), ARG1 (1:1,000, CST, 93668), tubulin (α-tubulin,1:10,000, EASYBIO, BE0031),Integrin αv (1: 1000, ABclonal, A19071), IBA1 (1:1000, Proteintech, 10904-1-AP), Integrin β1 (1:1000, ABclonal, A19072), Akt (1: 2000, ABclonal, A17909), Akt phosphorylation (Ser473) (1:1000, ABclonal, AP0637) overnight at 4°C. After three washes with TBST, the membranes were incubated with the HRP-linked secondary antibodies against horseradish peroxidase (HRP) anti-mouse IgG (CST, 7076), HRP anti-rabbit IgG (CST, 7074), HRP anti-goat IgG (EASYBIO, BE0103) in 5% milk for 1 h at room temperature. Signals on the membranes were developed with the HRP substrates luminol reagent (Millipore, WBKLS) and images were acquired by a molecular imager (BLT PHOTON TECHNOLOGY, GV6000PLUS) and analyzed by the GV6000 M2 software.

### Immunofluorescent staining

Immunofluorescent staining was performed in tissues. Mouse GBM xenografts were collected from mice after 4 weeks after the transplantation of GSC. Briefly, tumor sections were fixed in 4% PFA for 1 day and washed with PBS twice after that. Samples were blocked with a PBS solution containing 1% BSA plus 0.3% Triton X-100 for 30 min at room temperature, and then incubated with indicated primary antibody against CEBPB (1:1,000, Santa Cruz, sc-7962), CD163 (1:1,000, Abcam, ab182422), MRC1 (1:1,000, Abcam, ab64693), ARG1 (1:1,000, CST, 93668), IBA1 (1:1,000, Abcam, ab5076) overnight at 4°C followed by the fluorescent second antibody (Invitrogen, 1:1000) at room temperature for 2 h. Nuclei were counterstained with DAPI for 10 min, and then sections were mounted on glass and subjected to microscopy. ImageJ2 was used to quantify the positive cells.

### Multiplexed immunofluorescence assay

To visualize and assess the role of CEBPB-SPP1-Integrin αvβ1-Akt in M2 macrophage polarization within the tumor microenvironment, FFPE (Formalin-Fixed Paraffin-Embedded) slides from patient samples were analyzed using multiplex immunofluorescence and multispectral imaging techniques. This was conducted using a Absin Multiplex IHC kit (abs50029), specifically configured to detect SPP1 (Abcam, ab63856), CD163 (Abcam, ab182422), Integrin αvβ1 (Bioss bs-1356R), and phosphorylation Akt (Ser473) (CST, 4060). The staining procedure adhered to a rigorous protocol which included sequential incubation with primary and secondary antibodies, enhanced by tyramide signal amplification (TSA). Nuclei staining was performed with DAPI. The multispectral images were captured using the Mantra System (PerkinElmer).

### Cell viability assays

For cell viability assay, cell viability was determined at the indicated days after cell seeding using the Cell Counting Kit-8 (TargetMol, USA, C0005) Assay kit according to the manufacturer's protocol. To provide details, firstly, 2000 cells (U251 and A1207 cells) were plated into each well of a 96-well plate and the plate was incubated for 24 h for pre-cultivation. Afterward, 10 μL of CCK-8 solution was added to each well, and the plate was incubated in the incubator for another 3 h. Finally, the absorbance at 450 nm was measured using an enzyme-linked immunosorbent assay reader.

### Plasmid and lentiviral or RNAi transduction

Lentiviral plasmids for CEBPB shRNA knockdown (shCEBPB-59397, shCEBPB-59399), CEBPB overexpression and nonspecific control sequence (CON054) were purchased from Genechem (Shanghai, China). Lentiviral plasmid vector elements for CEBPB shRNA knockdown are hU6-MCS-CMV-Puromycin and for CEBPB overexpression are Ubi-MCS-3FLAG-SV40-BSD. Lentiviral particles were produced in 293T cells with PAX2 and PMD2G helper plasmids (Addgene) in DMEM medium. For lentiviral transduction, GBM cells were transducted with lentivirus expressing the shCEBPB, CEBPB overexpression and CON for 48 h, and then processed for next analysis. For RNAi transduction, RNAi-mediated knockdown of Integrin αv and Integrin β1, and their negative controls were all constructed by Synbio Technologies (Suzhou, China). The sequences of all siRNAs are listed in **Table S1**. Lipofectamine™ 3000 (Invitrogen, L3000015) was used as the transfection reagent.

### Animal experiments

All animal procedures were approved by the Animal Ethical and Welfare Committee of Tianjin Medical University Cancer Institute and Hospital (China, Ek2020157). The animal ethics approval number is AE-2022111. Mice used in these studies were 4 weeks old female mice. Nude mice (Beijing SiPeiFu Biotechnology Co., Ltd) were housed under a 12 h light/12 h dark cycle in a temperature (20-26°C) and humidity (30-70%) controlled environment and were fed ad libitum. In detail, firstly, the mice were anesthetized, and then they were secured on a stereotactic injection apparatus to perform the tumor implantation surgery. The nude mice's head is exposed in the field of view, and then a burr hole is drilled in the right cerebral cortex of the mice. Luciferase-expressing U251 (5×10^5^) or A1207 (5×10^4^) cells were transplanted into the right cerebral cortex of nude mice at a depth of 3.5mm. Finally, the incision is sutured closed. The size of orthotopic tumor was monitored by bioluminescence channel of IVIS Spectrum every week. The investigators were blinded to the group allocation and study outcome assessments of all mice.

### U937 monocyte Transwell and M2 polarization assays

U937 cells (ATCC) were cultured in the RPMI 1640 media 24 h before priming. U937 monocytes were primed with 100 nM Phorbol 12-myristate 13-acetate (PMA, Sigma) for 48 h to become monocyte-derived macrophages. Transwell assays assessing cell migration potential were performed on 24-well plates with inserts (BD Biosciences) according to the manufacturer's instruction. Briefly, 5×10^5^ primed U937 cells were cultured in the upper chamber and allowed to migrate for 24-48 h before fixation for crystal purple staining. Recombinant human SPP1 protein was purchased from R&DSystems (1433-OP-050/CF). Conditional media were obtained by culturing U251 and A1207 cells in DMEM media for 48 h and then used for the cell migration Transwell and M2 polarization assay. For the M2 polarization experiment with U937 cells, we cultured the U937 cells in conditioned medium for 48 h. Then, the cells were collected for the detection of M2 markers in subsequent experiments.

### Conditional media preparation

U251, A1207 cells were cultured in DMEM media and GBM727 was cultured in Neurobasal media for 48 h. Conditional media was collected from cultures at a density of 2×10^6^ cells/ml. The cells were removed by centrifugation (1000 rpm, 5 min), and the conditional media was sterile filtered through a 0.22 um filter (Biosharp, BS-PES-22). Then, the filtered conditioned medium is stored in a -80°C refrigerator.

### SPP1 ELISA

Secreted SPP1 from U251, A1207 and GBM727 cells and was measured using the Human Osteopontin (OPN) Quantikine ELISA Kit (R&D Systems, DOST00). To avoid differences in growth rates between different cells, supernatants were collected from 1×10^6^ cells after 12 h in culture and stored at -20°C for the assay. The plates were coated with mouse anti-human SPP1 overnight followed by blocking in reagent diluent (1% BSA in PBS). The supernatants and the standards were added in triplicate and incubated for 2 h at room temperature followed by a wash and incubation with the detection antibody and then with the horseradish peroxidase (HRP)-conjugated secondary antibody. Finally, the plates were incubated with the substrate solution, and the absorbance was measured at 450 nm (Thermo; 51119000). To determine the inhibitory concentration of the SPP1 inhibitor ASK8007 (Absin, abs171938) in GBM cells, we treated 1×10^6^ GBM727 CEBPB overexpressing cells with varying concentrations of ASK8007: 0, 150, 300, 500, and 1000 ng/ml. Two days later, the supernatants were collected and the concentration of SPP1 was measured.

### CUT&RUN and PCR assays

For CUT&RUN 62, 63, we used the Hyperactive pG-MNase CUT&RUN Assay Kit for PCR/qPCR (Nanjing Vazyme Biotech Co.,Ltd, HD101) and followed the instructions for the experiment. Briefly, we collected living U251 and A1207 cells (5×10^5^), washed them three times with PBS, and then counted the cells to take cells for the subsequent CUT&RUN experiment. We collected live cells, incubated the cells with ConA Beads Pro at room temperature for 10 min, and added the primary antibody (CEBPB: Santa Cruz Biotechnology, sc-7962; IgG: Millipore, 12-370) to the reaction solution overnight at 4℃. Perform MNase cleavage under 4°C to release the DNA fragments bound to the antibody. Finally, we collected and purified DNA fragments for subsequent PCR validation experiments. For PCR, we designed specific primers for the SPP1 promoter region (motif 1: forward 5′-GGCAGTGGCAGAAAACCT-3′ and reverse 5′-ACCAAGCCCTCCCAGAAT-3′; motif 2: forward 5′-AAAGGGTCGTATGGTTCA-3′ and reverse 5′-CTGTAGTTTACTCTGTGCC-3′). Perform PCR reaction on a thermal cycler (Applied Biosystems) and detect the amplification product through gel electrophoresis.

### Statistical analysis

All grouped data are presented as mean ± sem or mean ± sd. Significance between groups was analyzed by one-way ANOVA or Student's t-test. For Kaplan-Meier survival curves, statistical differences were determined by Wilcoxon or log-rank test. For correlation analysis, to address the issue of multiple comparisons, *p* values were adjusted for false discovery rate (FDR) using the Benjamini-Hochberg procedure. All analysis were carried out using Microsoft excel 2019, GraphPad Prism 8 and 9 software or R 4.0.5 and *p* < 0.05 was considered statistically significant. Detailed information is described in each figure legends. Except for the results from the public database, similar results were obtained from three independent experiments for all other results.

### Ethics approval

All animal procedures were approved by the Animal Ethical and Welfare Committee of Tianjin Medical University Cancer Institute and Hospital (China, Ek2020157). The animal ethics approval number is AE-2022111. All participants in the study provided their written consent in an informed manner.

## Supplementary Material

Supplementary figures, tables 1 and 3, methods.

Supplementary table 2.
