# Supplementary material for: The CEBPB+ glioblastoma subcluster specifically drives the formation of M2 tumor-associated macrophages to promote malignancy growth
Source: Theranostics. 2024 Jul 2;14(10):4107–26. doi: 10.7150/thno.93473 (PMC11234274; doi:10.7150/thno.93473)
Supplement: Supplementary file 1 — Supplementary figures, tables 1 and 3, methods. [file thnov14p4107s1.pdf]

Supplementary Information

Supplementary figures, tables and method

Supplementary figures

A

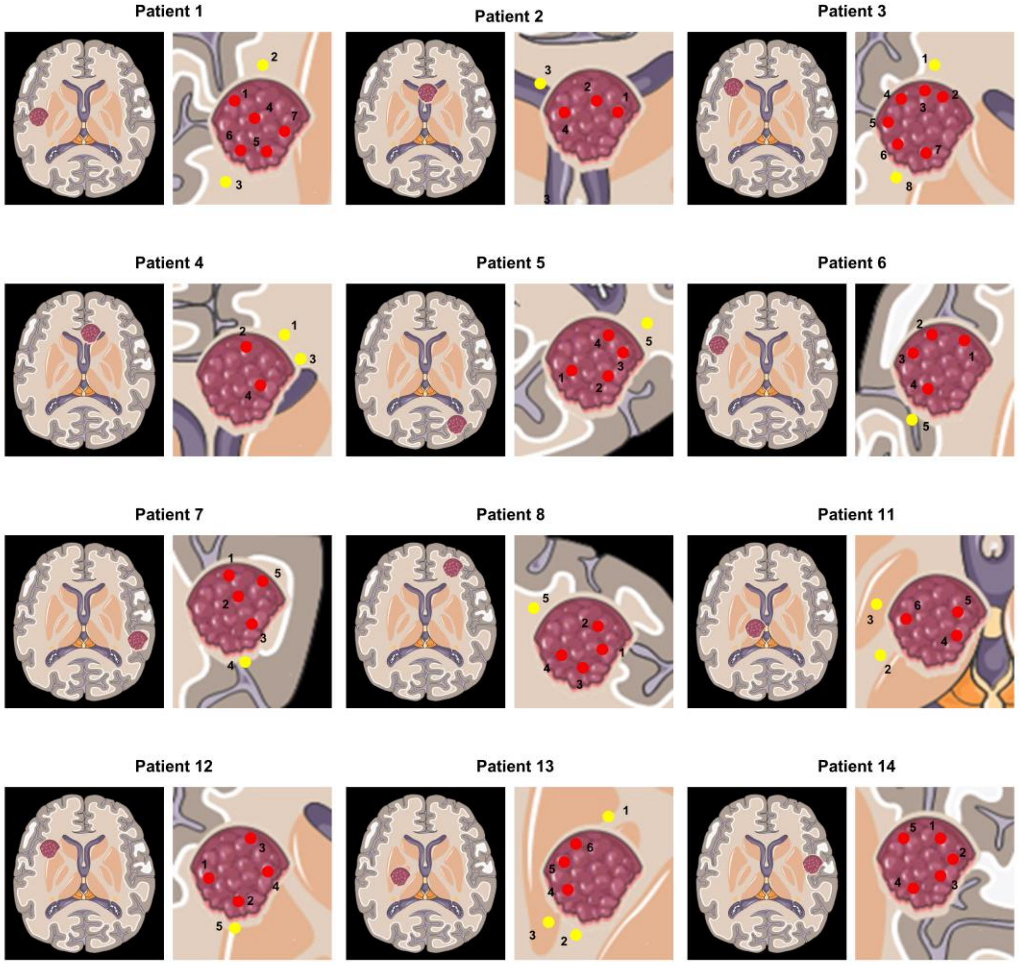

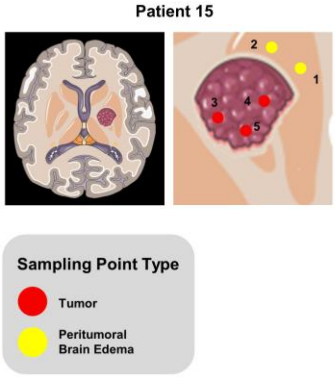

B

PatientID	WHO Grade	Histology	IDH1 R132 mutation	1p/19q co-del
CGGA_GS1	IV	GBM (O included)	-	NA
CGGA_GS2	IV	GBM	-	NA
CGGA_GS3	IV	GBM	-	NA
CGGA_GS4	IV	Gliosarcoma	NA	NA
CGGA_GS5	IV	GBM	-	NA
CGGA_GS6	IV	GBM (O included)	-	-
CGGA_GS7	III-IV	AOA-GBM	+	NA
CGGA_GS8	II	OA	+	NA
CGGA_GS9	II	OA	NA	NA
CGGA_GS11	IV	GBM	NA	NA
CGGA_GS12	IV	GBM	NA	NA
CGGA_GS13	IV	GBM (O included)	-	-
CGGA_GS14	II	O	+	NA
CGGA_GS15	NA	NA	NA	NA

Figure S1. Sampling region information for the 14 patients. (A) Medical imaging and biopsy locations of all sampling points in each patient based on MRI. Red and yellow dots mark locations of tumoral and peritumoral sampling points. **(B)** Pathology information of 14 patients collected in this study.

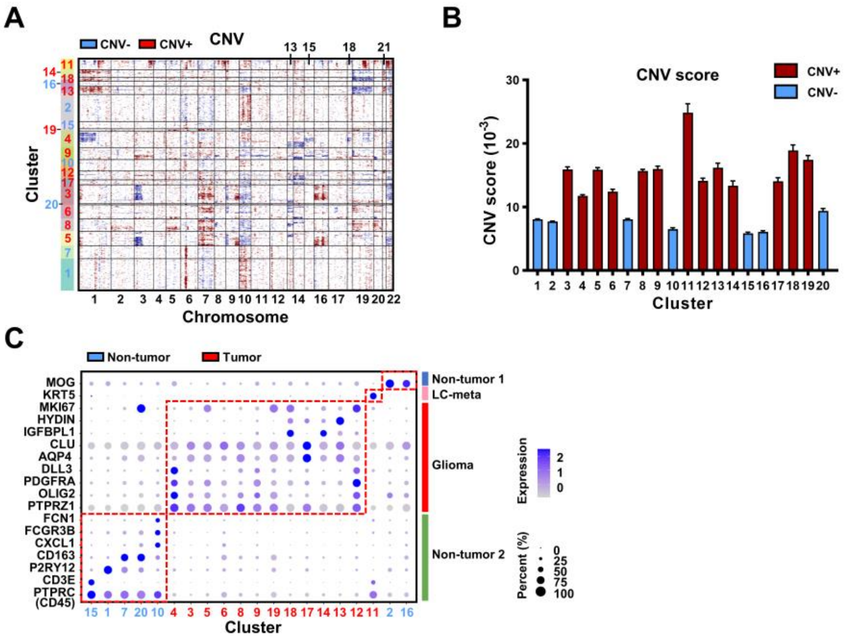

Figure S2. Identification of different clusters. (A) RNA-derived single-cell CNV information. Cell clusters were classified into CNV- and CNV+ categories, based on CNV information. **(B)** Quantification of CNV scores in cell clusters. CNV+ (red) or CNV- (blue). Data are represented as means ± s.e.m. **(C)** Dot plot was constructed according to cell cluster. With shared and specifically marker genes, clusters could be divided into non-tumor 1 (MOG+), non-tumor 2 (CD45+), regular glioma tumor (PTPRZ1+, OLIG2+ or AQP4+) and lung cancer metastasis cells (KRT5+).

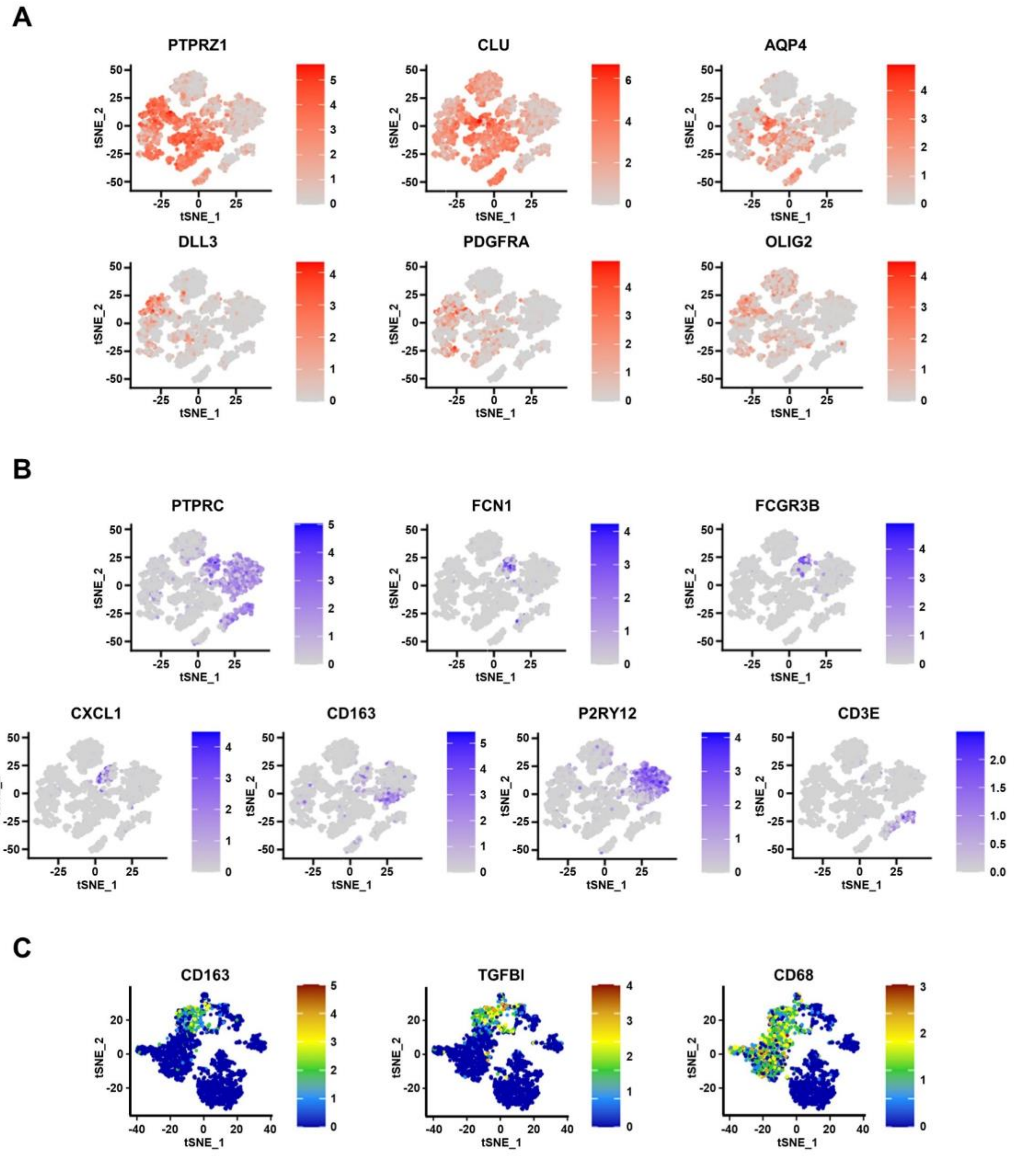

Figure S3. Patterns of marker gene expression. (A) Depicts a set of glioma clusters, whereas **(B)** signifies a collection of immune clusters within the entire cluster set. **(C)** Illustrates the M2 TAMs cluster within non-tumor cells.

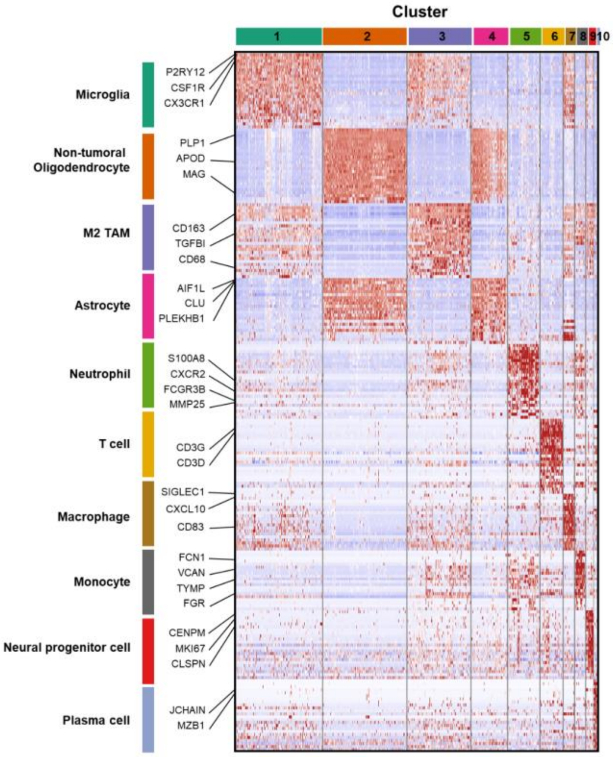

Figure S4. Heatmap shows marker genes of clusters of non-tumor cells.

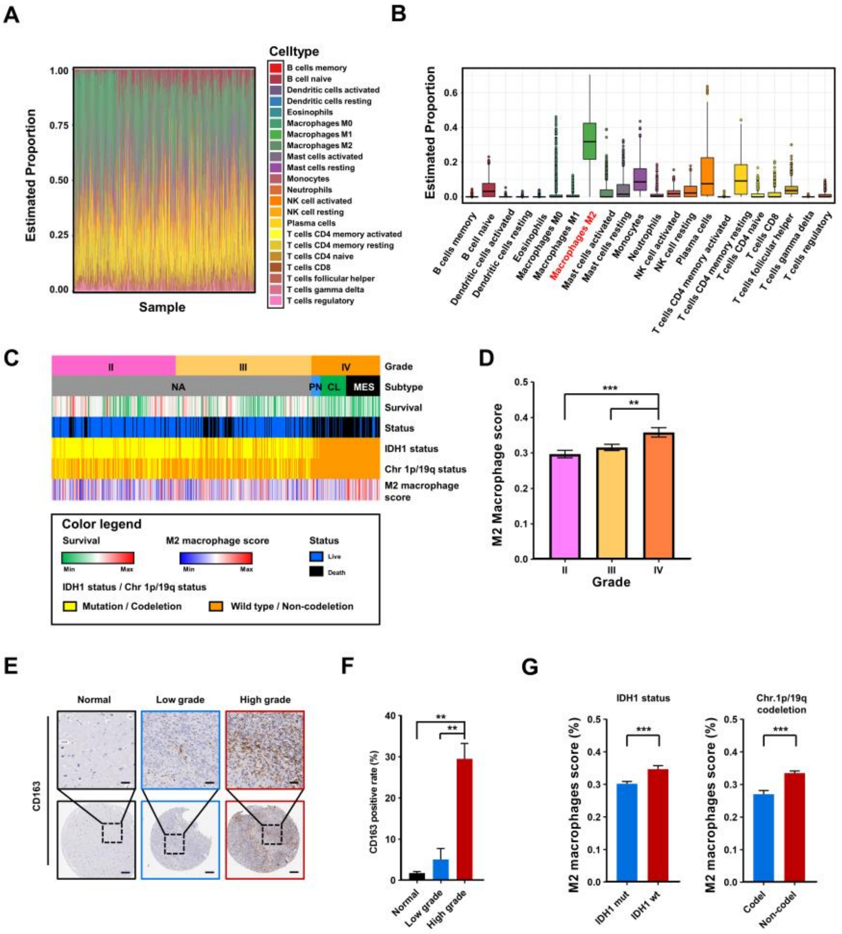

Figure S5. M2 TAMs are associated with poor prognosis in glioma patients. Based on the TCGA GBMLGG database, **(A)-(B)** demonstrated the immune infiltration status of 22 immune cell types. Data are expressed as means and interquartile range (25-75 percentile). **p* < 0.05, ***p* < 0.01, ****p* < 0.001, ns: *p* > 0.05; two-tailed unpaired t-test. **(C)-(D)** shows the M2 macrophages infiltration score in glioma patients of different grades (n = 222, Grade II; n = 243, Grade III; n = 122; Grade IV). Data are represented as means ± s.e.m. ***p* < 0.01, ****p* < 0.001; two-tailed unpaired t-test. **(E)** Representative immunohistochemistry (IHC) of M2 macrophages marker (CD163) in glioma patients of different grades in The Human Protein Atlas. Boxed areas are further magnified. Scale bars, 100 µm (up) and 25 um (down). **(F)** Quantification of CD163 positivity rate in **(E)** using ImageJ. n = 3 (normal), n = 4 (Low grade) and n = 14 (High grade) biological independent tumor samples. Data are represented as means ± s.e.m. ***p* < 0.01; one-way ANOVA with Tukey's method for multiple comparisons. **(G)** shows the M2 macrophages infiltration score in glioma patients with IDH1 status (IDH1 mutation or IDH1 wild type) and chromosome 1p/19q status (codeletion or non-codeletion) in the TCGA GBMLGG database (n = 386, IDH1 mutation; n = 201, IDH1 wild type; n = 156, chromosome 1p/19q codeletion; n = 431, chromosome 1p/19q non-codeletion). Data are represented as means ± s.e.m. ****p* < 0.001; two-tailed unpaired t-test.

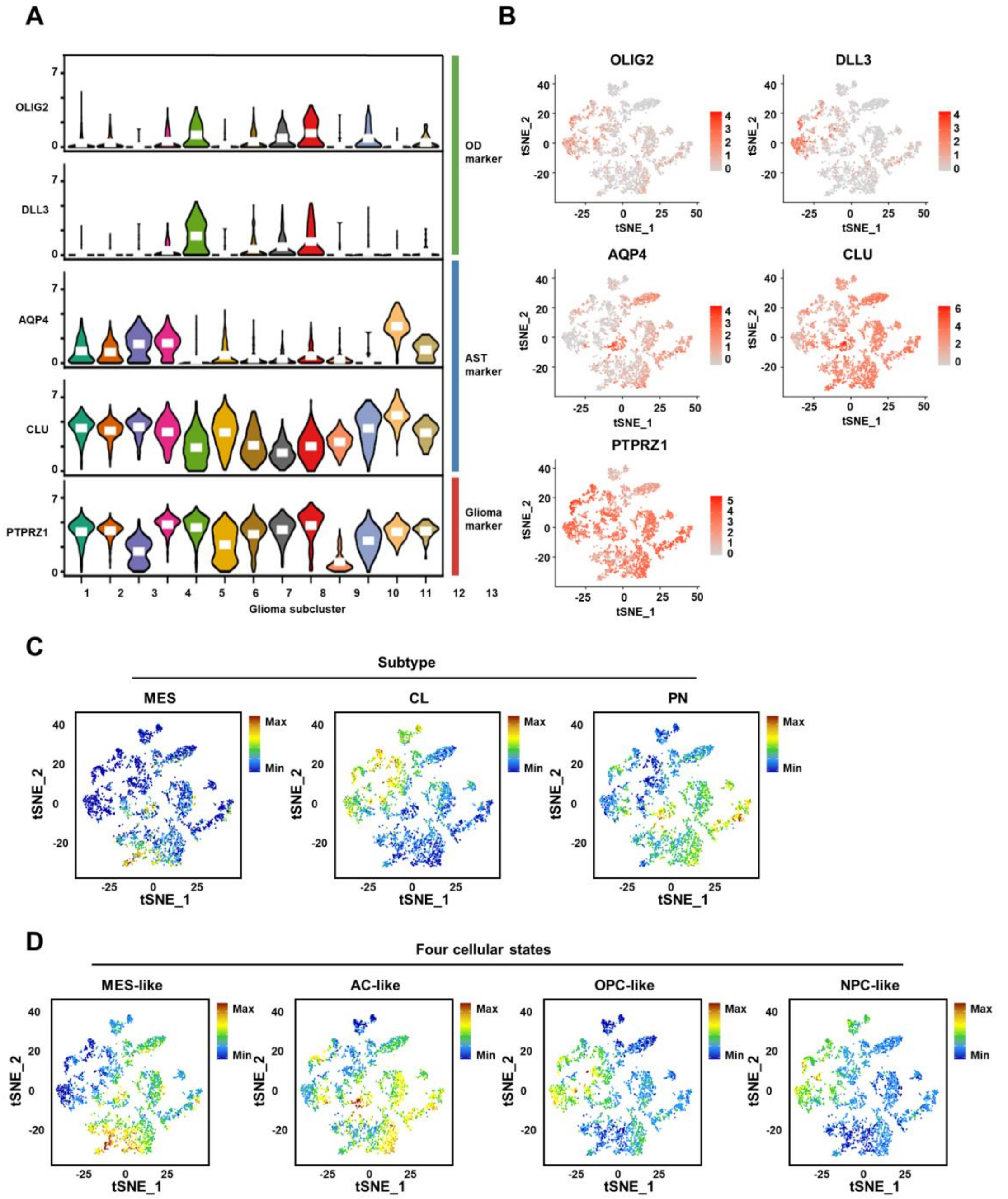

Figure S6. (A)-(B) display represented marker genes for Oligodendroglioma (OD: OLIG2, DLL3), Astrocytoma (AST: AQP4, CLU), glioma (PTPRZ1) in all glioma cells. The t-SNE plot shows the enrichment of 3 subtypes **(C)** and 4 cellular states **(D)**.

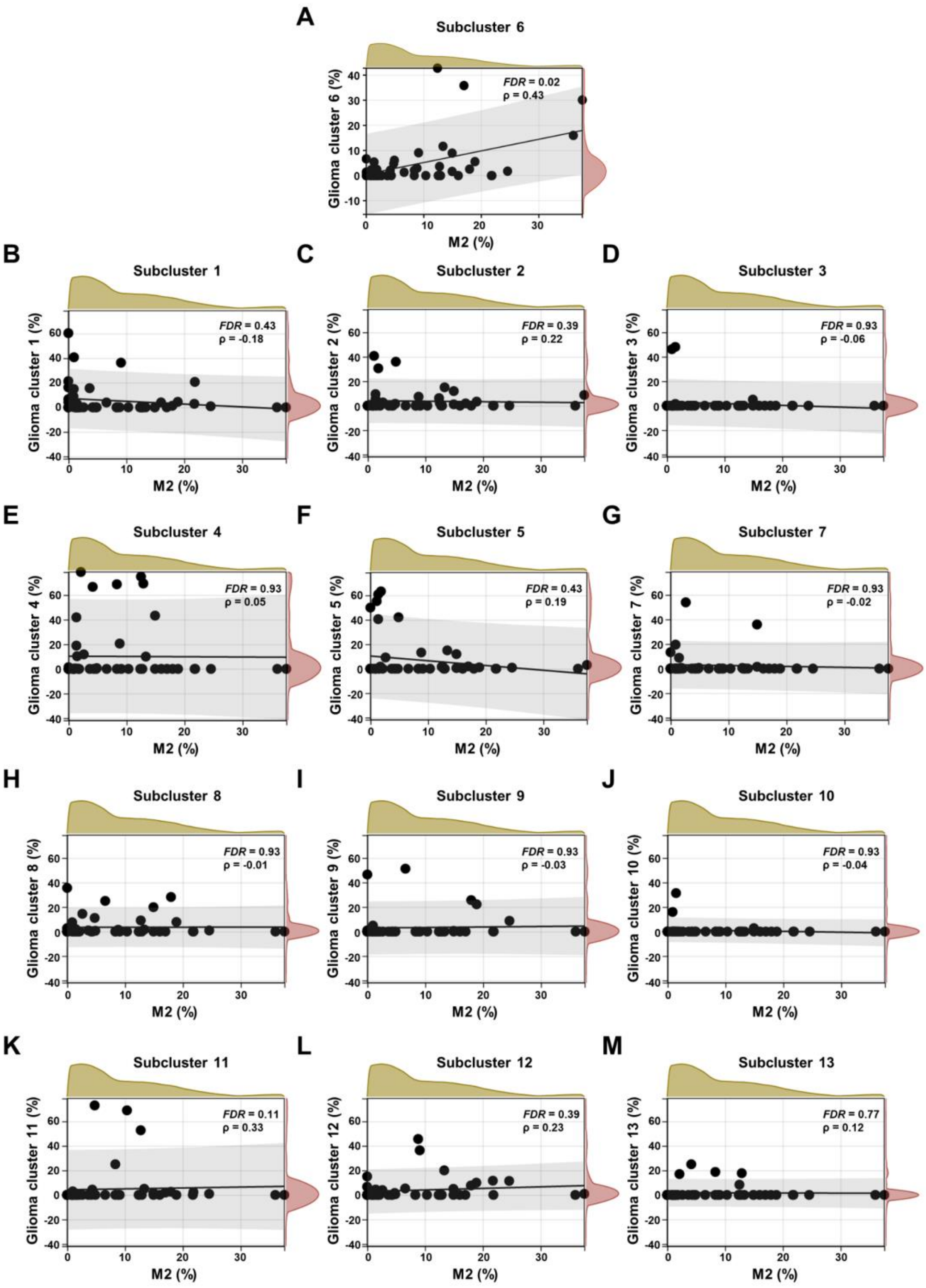

Figure S7. Correlation analysis of 13 glioma subclusters with M2 TAMs. (A)-(M) Spearman's correlation analysis of each glioma cluster proportion (%) and M2 macrophage proportion (%) in 51 tumor regions.

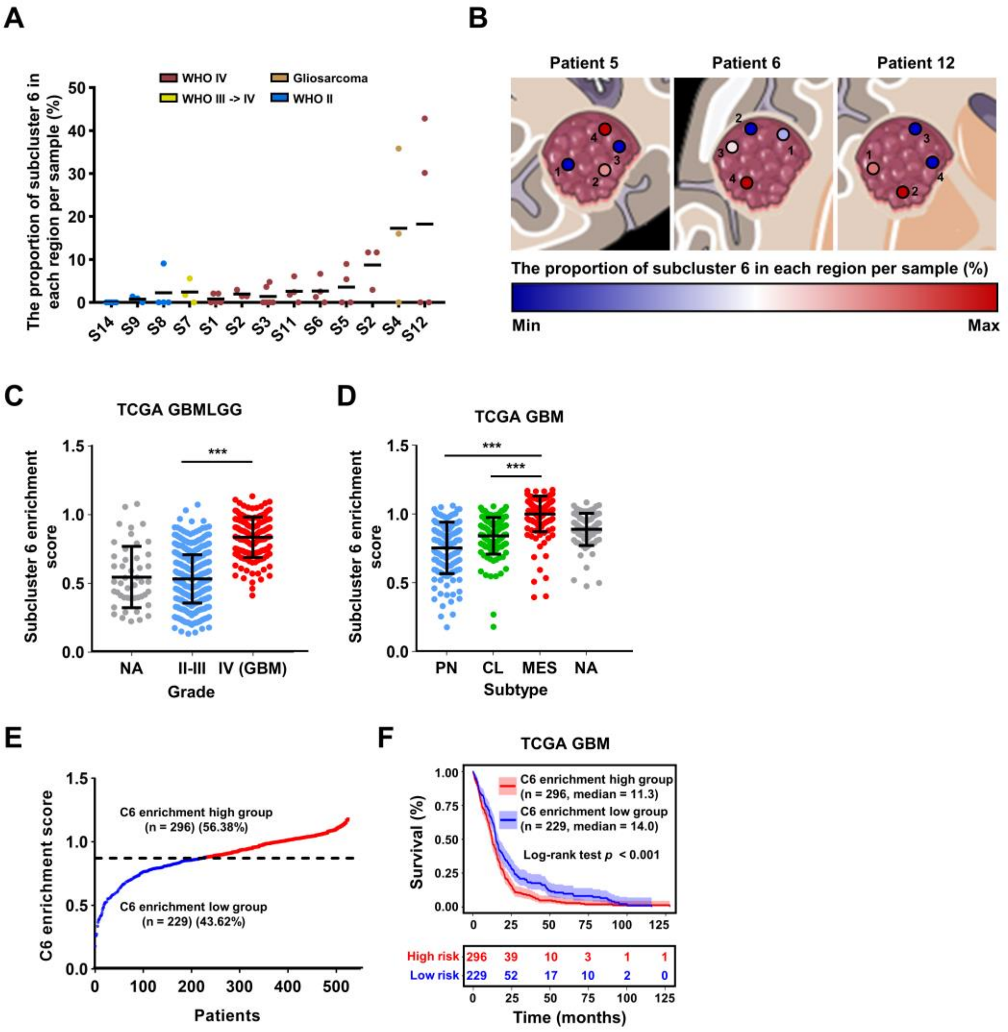

Figure S8. The cluster 6 is prevalent in a majority of GBM patients with poorer prognoses. (A) The scatter plot displays the proportion of subcluster 6 in each region per sample (%) with each spots colored according to the WHO grade of the sample. **(B)** The proportion of subcluster 6 in each region is displayed for representative patients. **(C)** The subcluster 6 enrichment score in grade (n = 467, II-III; n = 149, IV; n = 47, NA) from the TCGA GBM database. Black bars indicate mean ± s.d. ****p* < 0.001 ; one-way ANOVA with Tukey's method for multiple comparisons. **(D)** The subcluster 6 enrichment score in subtype (n = 137, proneural (PN); n = 144, classical (CL); n = 156, mesenchymal (MES); n = 88, NA) from the TCGA GBM database. Black bars indicate mean ± s.d. ****p* < 0.001 ; one-way ANOVA with Tukey's method for multiple comparisons. **(E)** GBM patients are categorized into two groups, high and low, based on the mean value of the enrichment score for subcluster 6. Among them, the high group represents 56.38% of the GBM patients, while the low group accounts for 43.62%. **(F)** Kaplan–Meier curves of GBM patient survival stratified by the mean value of subcluster 6 enrichment score from TCGA GBM databases. *P* values were determined by log-rank.

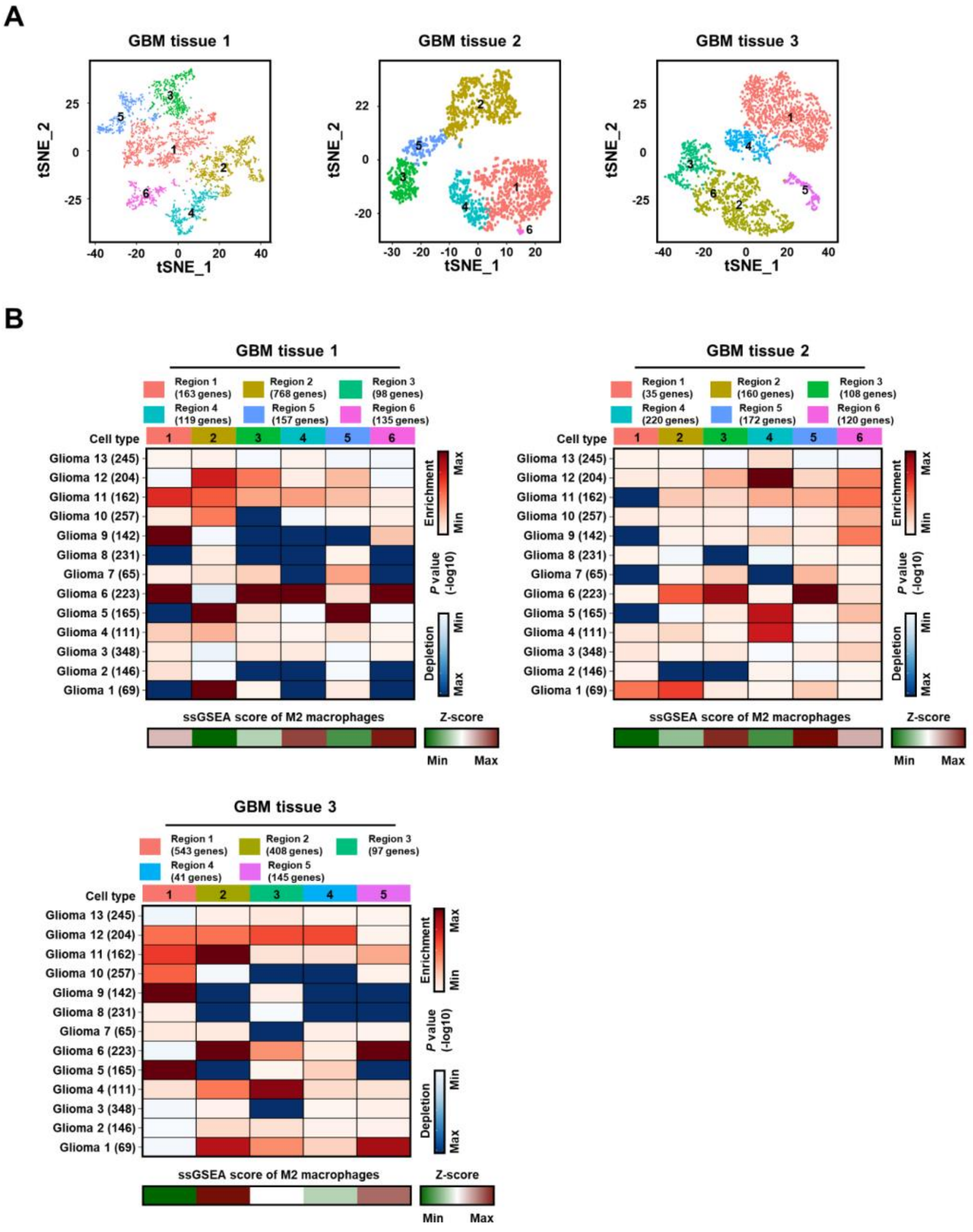

Figure S9. Single-cell RNA and Spatial transcriptomics information. (A) T-SNE clustering analysis of GBM tissue 1, GBM tissue 2, GBM tissue 3 in the ST region. **(B)** MIA analysis of GBM tissue 1, GBM tissue 2, GBM tissue 3. Red indicates enrichment, while blue represents depletion. Below, the ssGSEA enrichment scores for M2 macrophages in different regions are displayed.

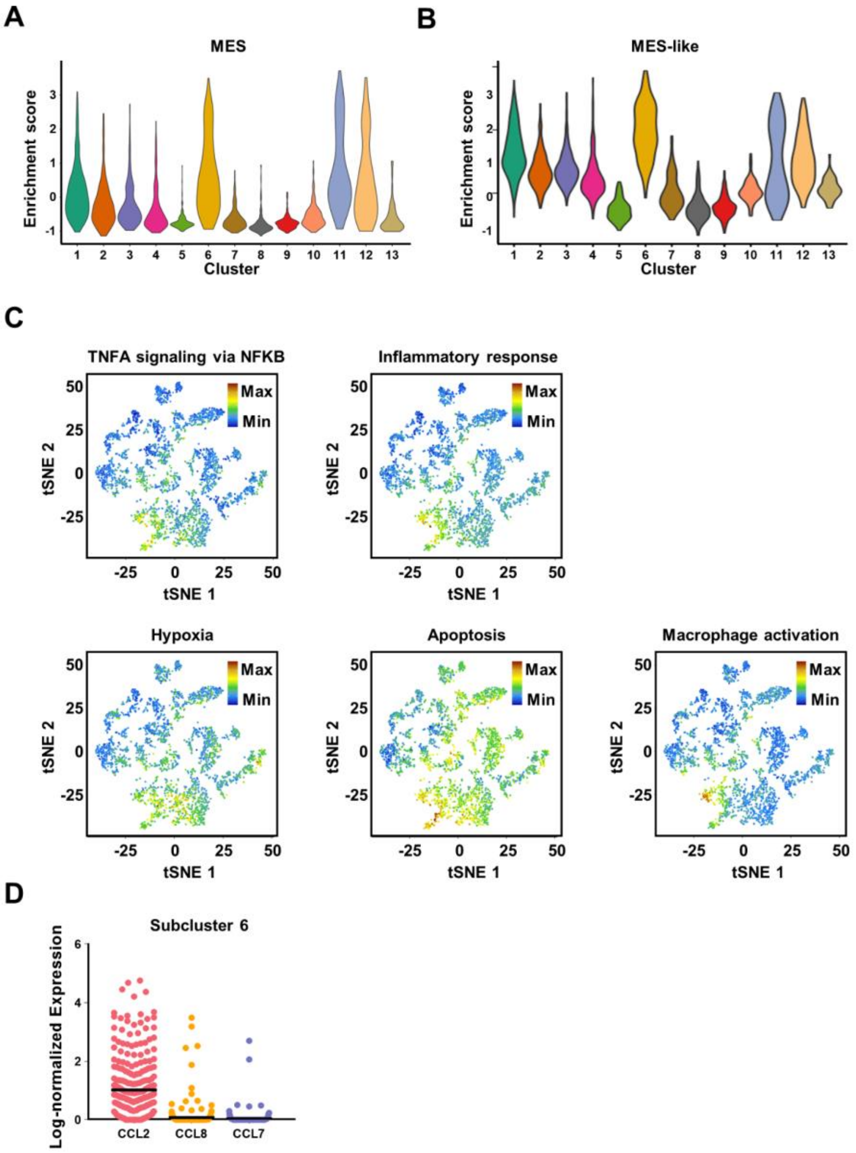

Figure S10. The expression profile of the MES subtype **(A)** and MES-like states **(B)** of GBM are displayed. **(C)** The feature plot displays the differential expression of signaling pathways across all glioma cells. **(D)** Expression levels of monocyte chemoattractant proteins (CCL2, CCL7, CCL8) in subcluster 6.

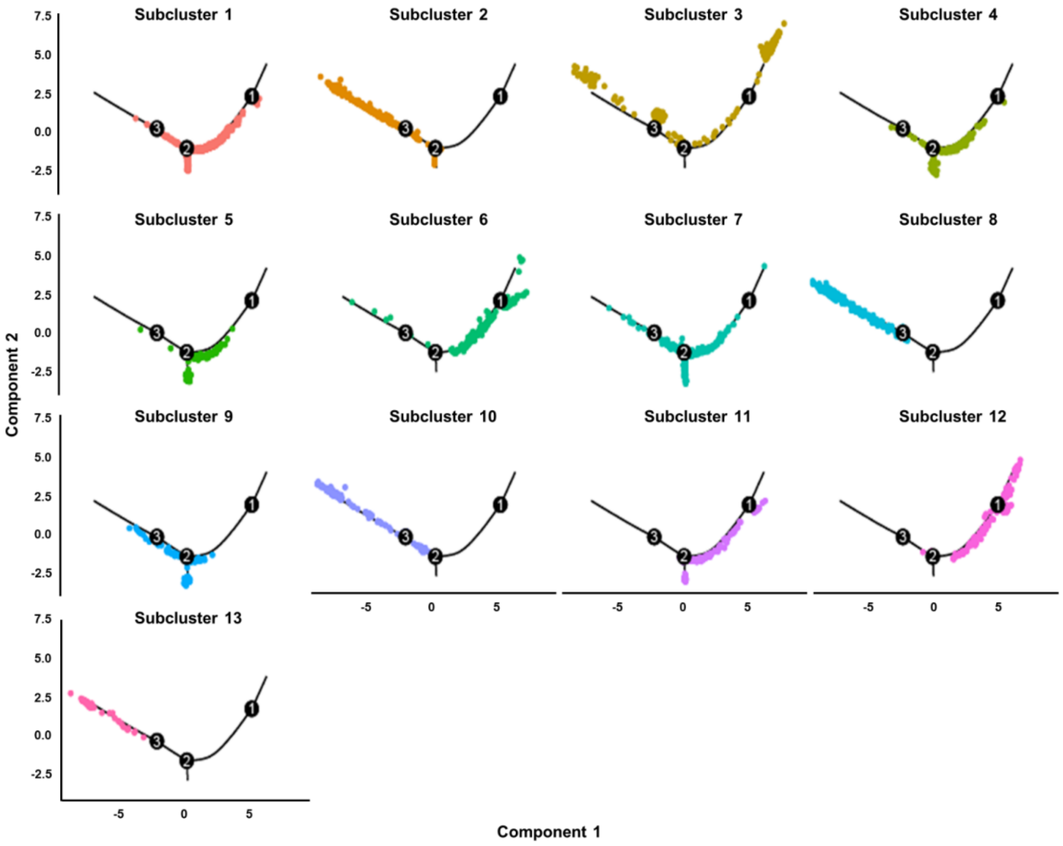

Figure S11. Trajectory analysis shows the distribution of each glioma cluster, respectively.

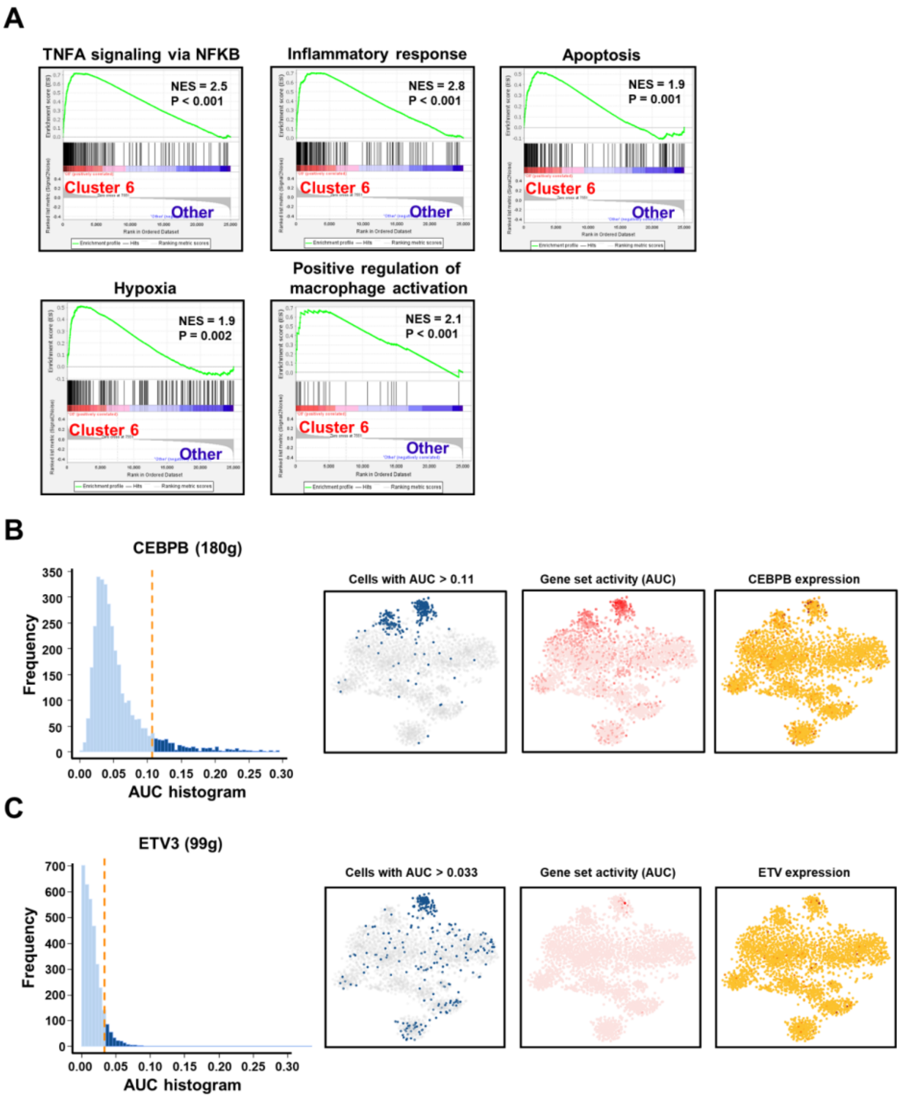

Figure S12. The pathway enrichment status of GBM cluster 6. (A) Gene set enrichment analysis (GSEA) shows functional enrichment in GBM cluster 6. **(B)-(C)** CEBPB-regulons and ETV-regulons enriched expression pattern on binary regulon activity are shown.

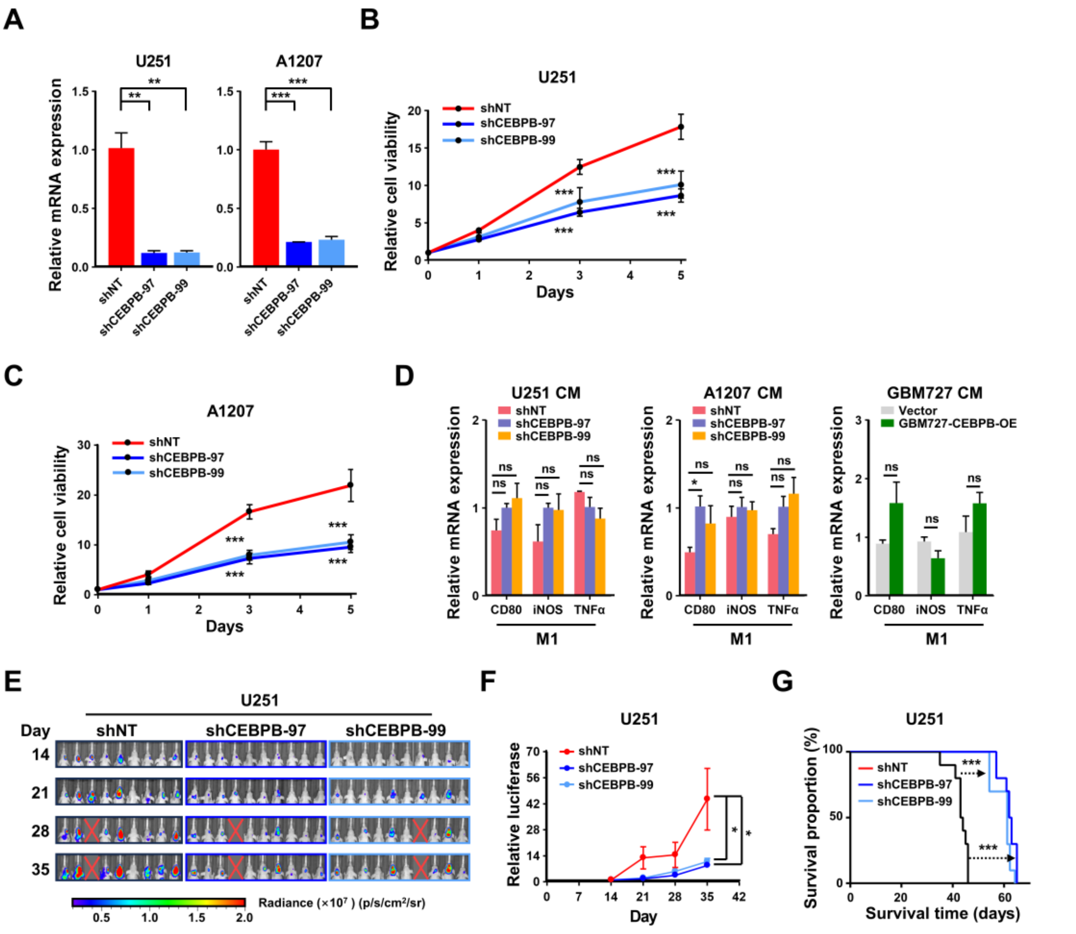

Figure S13. CEBPB promotes malignant progression of GBM cells in vitro. (A) Relative mRNA expression of CEBPB in GBM cells (U251 and A1207) transduced with non-targeting shRNA (shNT) or CEBPB shRNA (shCEBPB) through lentiviral infection. Data are represented as means ± s.e.m. n = 3 independent experiments. **$p < 0.01$, ***$p < 0.001$. Statistical significance was determined by one-way ANOVA analysis. **(B)-(C)** Cell viability assay of GBM cells transduced with shNT or shCEBPB. n = 6 (U251) or n = 6 (A1207) biological independent samples. Data are shown as means ± s.e.m.***$p < 0.001$, two-way ANOVA analysis followed by Tukey's multiple test. **(D)** qPCR was used to detect the expression of M1 markers (iNOS, TNFα and CD80) in monocytes treated with 100ng/ml PMA and GBM CM for 72 h. Data are represented as means ± s.e.m. n = 3 independent experiments. *$p < 0.05$, ns: $p > 0.05$. **(E)-(G)** Left, representative images on day 14, 21, 28, 35 post transplantation are shown; bioluminescence is measured in p/s/cm^2/sr. Middle, quantification of relative luciferase signals during 35 days. U251: shNT (n = 8), shCEBPB-97 (n = 9), shCEBPB-99 (n = 9); Data are represented as means ± s.e.m. *$p < 0.05$, one-way ANOVA with Tukey's method for multiple comparisons. Right, Kaplan–Meier survival curves of mice bearing U251-derived xenografts expressing shNT or shCEBPB. ***$p < 0.001$, log-rank test. U251: shNT (n = 10), shCEBPB-97 (n = 10), shCEBPB-99 (n = 10).

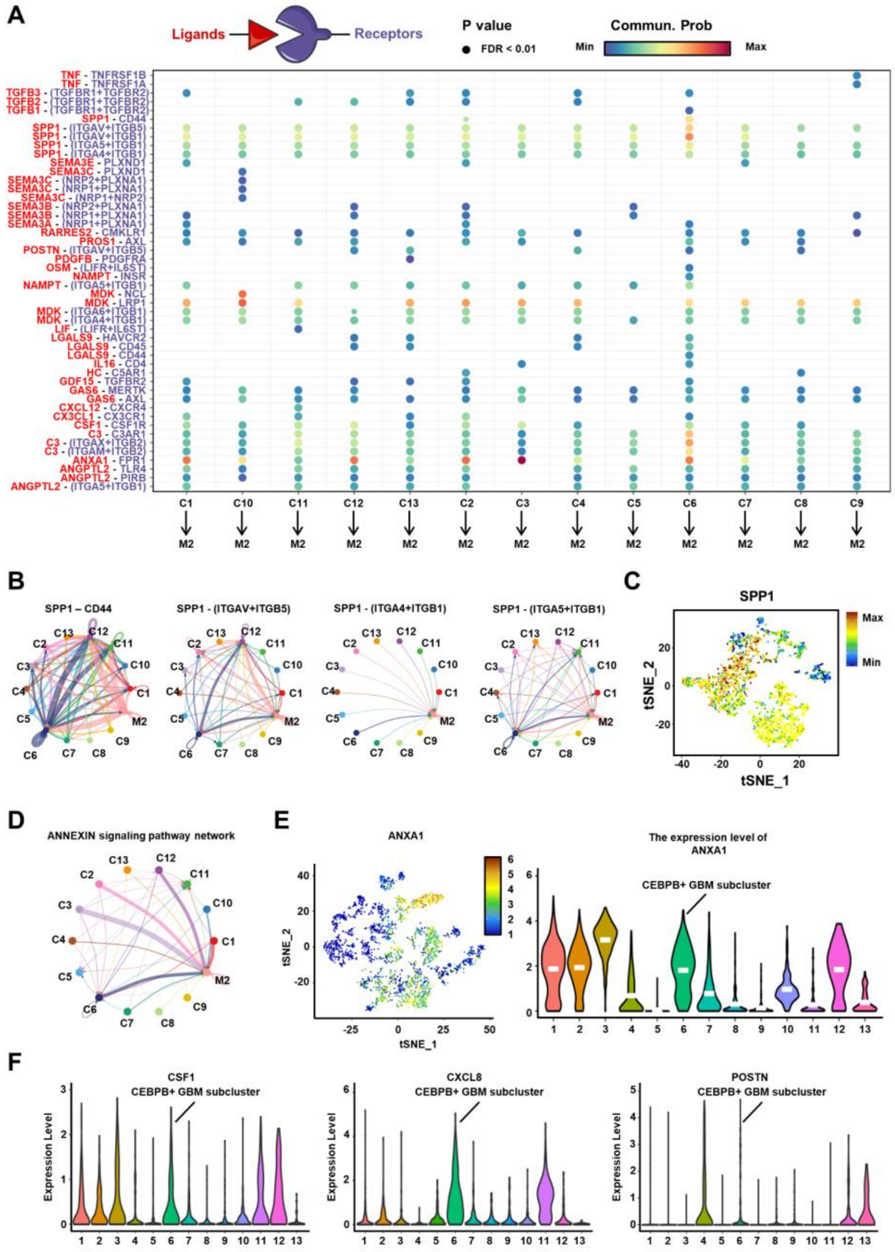

Figure S14. Cell communication between M2 TAMs and different glioma cluster. **(A)** All the significant ligand-receptor pairs that contribute to the signaling sending from 13 glioma clusters to M2 TAMs. The dot color and size represent the calculated communication probability and *p*-values. *p*-values are computed from one-sided permutation test. **(B)** The inferred SPP1 – CD44, SPP1 - (ITGAV+ITGB5), SPP1 - (ITGA4+ITGB1) and SPP1 - (ITGA4+ITGB1) interaction network. Circle sizes are proportional to the number of cells in each cell cluster and edge width represents the communication probability. **(C)** The expression distribution of SPP1 in non-tumor cells. **(D)** The inferred ANNEXIN signaling pathway network. **(E)** The expression distribution of ANXA1 on t-SNE coordinates (left) and their expression in various glioma clusters (right). **(F)** The violin plot displays the expression of CSF1, CXCL8, and POSTN, which have been reported to induce M2 polarization in macrophages, across 13 glioma subclusters.

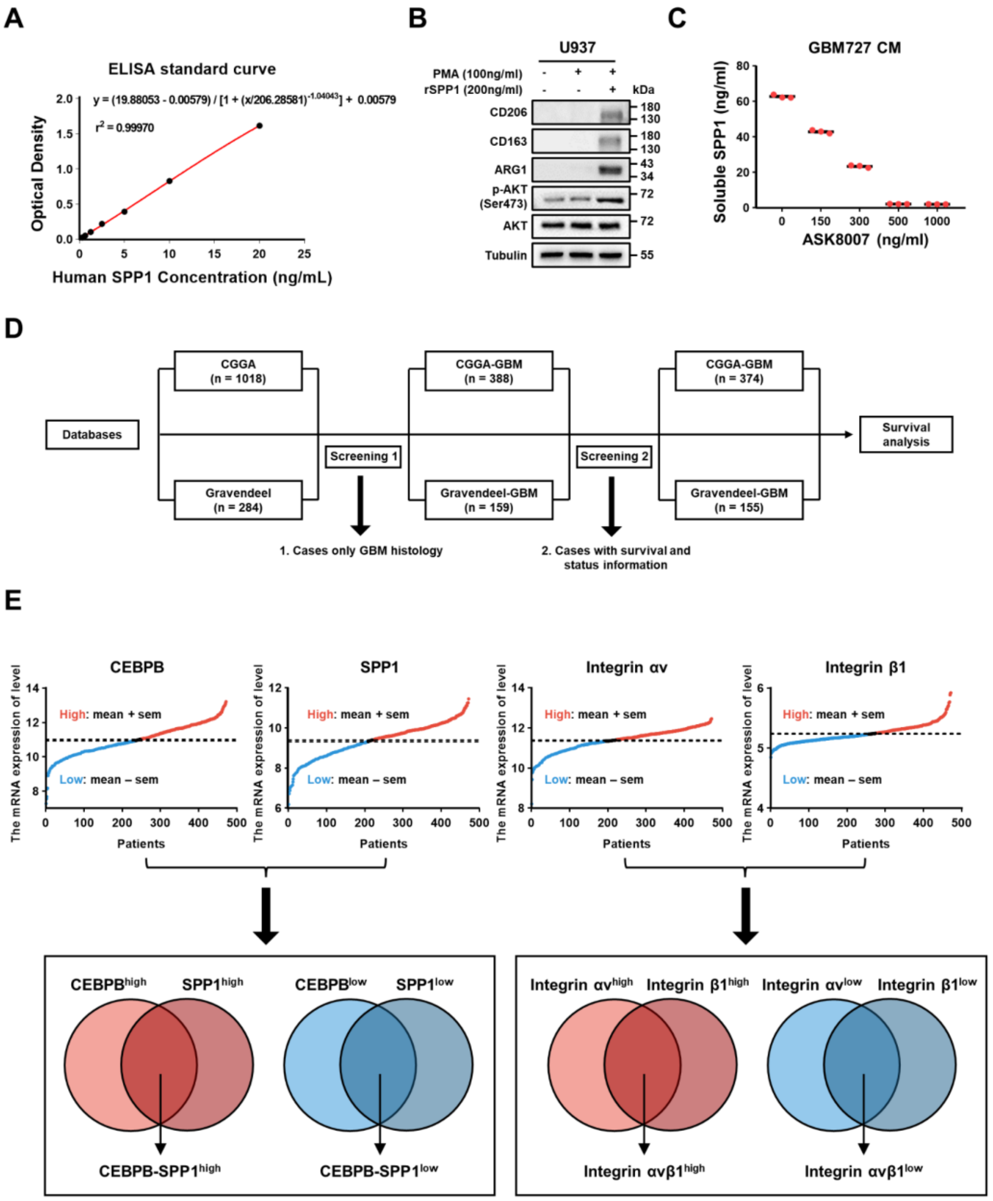

Figure S15. Patient information from the database. (A) The ELISA standard curve of SPP1. **(B)** Immunoblot analysis of M2 macrophages markers (CD206, CD163 and ARG1) and Akt phosphorylation (Ser473) in monocytes (U937 cells) treated with 100 ng/ml PMA and 200 ng/ml rSPP1 protein for 72 h. Akt and α-tubulin were blotted as the loading control. **(C)** ELISA was used to measure the inhibitory effects of different concentrations of ASK8007 on SPP1. **(D)** Patient screening criteria for the survival analysis in CGGA and Gravendeel databases. **(E)** Classification criteria for the patients in public databases. Patients with gene expression levels greater than the mean + SEM were classified as the high group, while those with levels lower than the mean - SEM were classified as the low group. Patients who are both in the CEBPB^{high} group and the SPP1^{high} group are classified as the CEBPB-SPP1^{high} group. Similarly, those who are both in the CEBPB^{low} group and the SPP1^{low} group are classified as the CEBPB-SPP1^{low} group. The grouping for Integrin αvβ1 follows the same criteria as mentioned above.

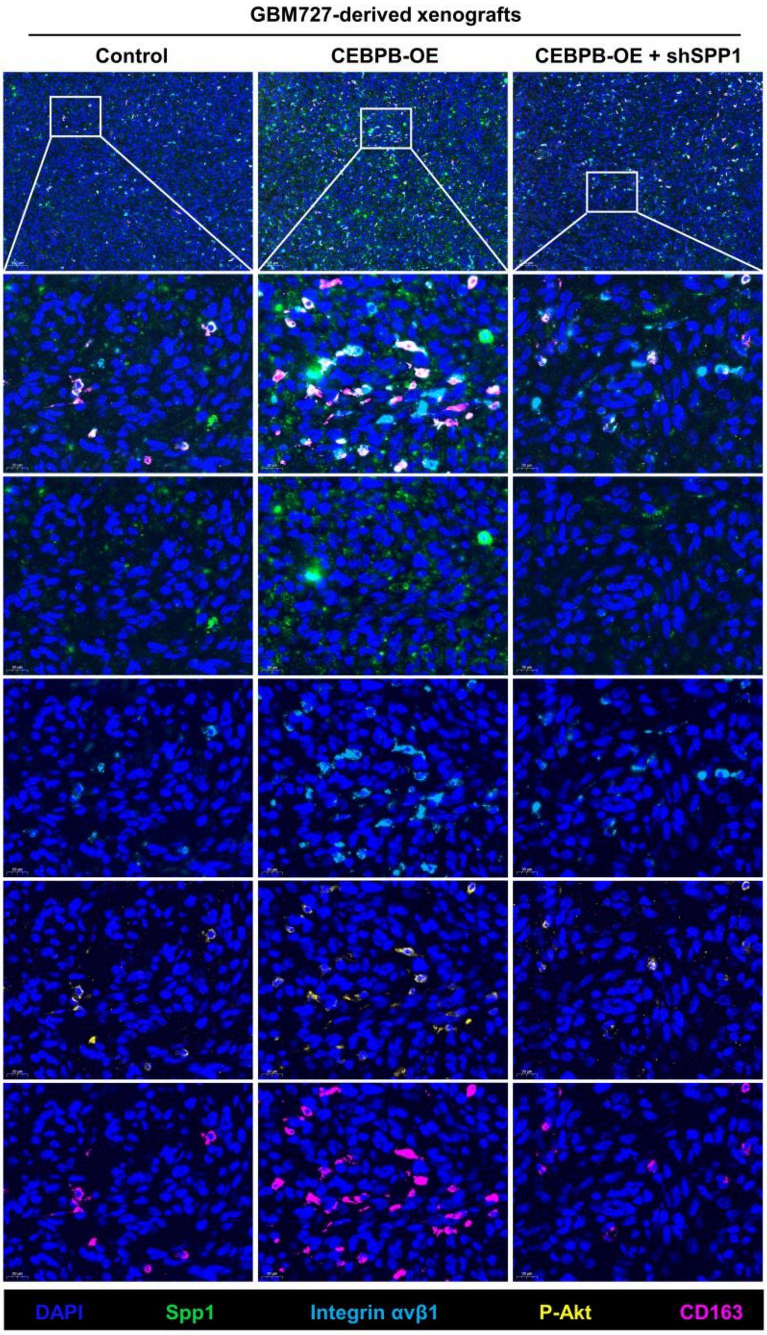

Figure S16. Representative magnified images from multiplex immunofluorescence show the relative cell number of SPP1^{+} Integrin avβ1^{+} CD163^{+} P-Akt^{+} M2 TAMs in GBM727.

Supplementary tables

Table S1. RNAi sequence

Gene Symbol	Sequence	
	Sense (5'-3')	Antisense (5'-3')
Hs-ITGAV-si-1	GACAAACUCACUCCAAUUAdTdT	UAAUUGGAGUGAGUUUGUCdTdT
Hs-ITGAV-si-2	GAUCGAGCUAUCUUAUACAdTdT	UGUAUAAGAUAGCUCGAUCdTdT
Hs-ITGB1-si-1	GGAACCCUUGCACAAGUGAdTdT	UCACUUGUGCAAGGGUUCCdTdT
Hs-ITGB1-si-2	GGAUAUUACUCAGAUCCAAdTdT	UUGGAUCUGAGUAAUAUCCdTdT

Table S2. scRNA_13_giloma_cluster_allmarkergene

This table is too large to be presented here. Therefore, please refer to the separate Table S2 for detailed information.

Table S3. C6 interacts with macrophages

source	target	ligand	receptor	prob	pval	interaction_name
C6	M2	ANXA1	FPR1	0.095979946	0	ANXA1 - FPR1
C6	M2	SPP1	ITGAV_ITGB1	0.088686273	0	SPP1 - (ITGAV+ITGB1)
C6	M0	C3	ITGAX_ITGB2	0.087813966	0	C3 - (ITGAX+ITGB2)
C6	M0	C3	C3AR1	0.087707451	0	C3 - C3AR1
C6	M2	C3	ITGAX_ITGB2	0.079967038	0	C3 - (ITGAX+ITGB2)
C6	M2	C3	C3AR1	0.063869734	0	C3 - C3AR1
C6	M2	SPP1	ITGAV_ITGB5	0.057493575	0	SPP1 - (ITGAV+ITGB5)
C6	M2	MDK	LRP1	0.049939893	0	MDK - LRP1
C6	M2	SPP1	CD44	0.045254967	0	SPP1 - CD44
C6	M2	C3	ITGAM_ITGB2	0.04457857	0	C3 - (ITGAM+ITGB2)
C6	M2	SPP1	ITGA5_ITGB1	0.038178321	0	SPP1 - (ITGA5+ITGB1)
C6	M0	C3	ITGAM_ITGB2	0.031103896	0	C3 - (ITGAM+ITGB2)
C6	M2	SPP1	ITGA4_ITGB1	0.01874675	0	SPP1 - (ITGA4+ITGB1)
C6	M2	CSF1	CSF1R	0.015606687	0	CSF1 - CSF1R
C6	M2	NAMPT	ITGA5_ITGB1	0.013985613	0	NAMPT - (ITGA5+ITGB1)
C6	M0	ANGPTL2	TLR4	0.007080792	0	ANGPTL2 - TLR4
C6	M2	MDK	ITGA6_ITGB1	0.006209335	0	MDK - (ITGA6+ITGB1)

C6	M0	PROS1	AXL	0.006027493	0	PROS1 - AXL
C6	M2	MDK	ITGA4_ITGB1	0.005436138	0	MDK - (ITGA4+ITGB1)
C6	M2	LGALS9	PTPRC	0.003993861	0	LGALS9 - CD45
C6	M2	PROS1	AXL	0.00384002	0	PROS1 - AXL
C6	M0	LGALS9	HAVCR2	0.003303798	0.02	LGALS9 - HAVCR2
C6	M0	RARRES2	CMKLR1	0.002953039	0	RARRES2 - CMKLR1
C6	M2	ANGPTL2	TLR4	0.002777832	0	ANGPTL2 - TLR4
C6	M0	GAS6	AXL	0.002728528	0	GAS6 - AXL
C6	M2	LGALS9	HAVCR2	0.002252528	0	LGALS9 - HAVCR2
C6	M0	GAS6	MERTK	0.002188856	0	GAS6 - MERTK
C6	M2	ANGPTL2	ITGA5_ITGB1	0.002071452	0	ANGPTL2 - (ITGA5+ITGB1)
C6	M2	GAS6	AXL	0.001736863	0	GAS6 - AXL
C6	M2	GAS6	MERTK	0.001215757	0	GAS6 - MERTK
C6	M2	IL16	CD4	0.001076989	0	IL16 - CD4
C6	M2	NAMPT	INSR	0.0008065	0	NAMPT - INSR
C6	M2	LGALS9	CD44	0.000672032	0	LGALS9 - CD44
C6	M2	GDF15	TGFBR2	0.000640687	0	GDF15 - TGFBR2
C6	M2	TGFB3	TGFbR1_R2	0.000539678	0	TGFB3 - (TGFBR1+TGFBR2)
C6	M0	GDF15	TGFBR2	0.00052361	0	GDF15 - TGFBR2
C6	M0	CX3CL1	CX3CR1	0.000442944	0	CX3CL1 - CX3CR1
C6	M0	TGFB3	TGFbR1_R2	0.000421211	0	TGFB3 - (TGFBR1+TGFBR2)
C6	M2	CX3CL1	CX3CR1	0.000329473	0	CX3CL1 - CX3CR1
C6	M2	ANGPTL2	LILRB3	0.000322107	0	ANGPTL2 - PIRB
C6	M2	RARRES2	CMKLR1	0.000304666	0	RARRES2 - CMKLR1
C6	M2	SEMA3A	NRP1_PLXNA1	0.000237792	0	SEMA3A - (NRP1+PLXNA1)
C6	M2	OSM	LIFR_IL6ST	0.000179623	0	OSM - (LIFR+IL6ST)
C6	M2	POSTN	ITGAV_ITGB5	0.0000869	0	POSTN - (ITGAV+ITGB5)
C6	M2	TGFB1	TGFbR1_R2	0.0000412	0	TGFB1 - (TGFBR1+TGFBR2)
C6	M0	FGF1	FGFR2	3.09E-05	0	FGF1 - FGFR2
C6	M0	FGF2	FGFR2	1.64E-05	0	FGF2 - FGFR2
C6	M0	POSTN	ITGAV_ITGB5	1.57E-05	0	POSTN - (ITGAV+ITGB5)

Supplementary method

scRNA-seq data process

The scRNA-seq data of GSE117891 were explored using the R package Seurat (version 4.0.5). The first step was to filter out low quality cells with a cutoff value of less than 200 total feature RNA and genes that were expressed in less than three cells. Ultimately, we obtained 6,148 high-quality cells for downstream analysis. To reduce dimensions, principal component analysis (PCA) was performed (npcs = 30) and followed by t-distributed stochastic neighbor embedding (tSNE) algorithms (dims = 1:10). Then, 20 cell clusters were found in the first place after applying the FindNeighbors and FindClusters functions. The resolution parameter in the find clusters procedure was set to 1.0 by multiple trial and observation. The genes differentially expressed (DE) in each cluster were identified using FindAllMarkers function (only.pos = TRUE, min.pct = 0.25 and logfc.threshold = 0.5). For these cells, we use a t-test between each of 2 groups of cells; genes that met the condition of $\log_2 FC > 1$ and p value < 0.01 were kept as significant DE genes. Then, we manually annotated each cell cluster based on marker genes specific to each cluster. 2587 immune cells (immune cluster) and 3312 glioma cells (glioma cluster, T-Cilium cluster, proliferation cluster) were further divided into 10 subclusters (resolution = 0.3) and 13 clusters (resolution = 0.5), respectively. Signature scores of cells were performed with AddModuleScore function.

Spatial transcriptomics data processing

The spatial transcriptomic (ST) data of glioma were analyzed in R using the Seurat 4.0 package according to the recommended data processing guidelines (https://satijalab.org/seurat/articles/spatial_vignette.html). In summary, we performed data normalization using the SCTranform function, followed by dimensionality reduction with PCA and tSNE algorithms. Clustering was then performed using the default resolution of the first 30 principal components. Then, we employed the MIA method to perform joint analysis of the scRNA-seq data and spatial transcriptomics data.

Multimodal intersection analysis (MIA)

To integrate the scRNA-seq and spatial transcriptomic (ST) datasets, we employed the MIA approach. This involved identifying sets of genes specific to each cell cluster and tissue region, and then measuring the degree of their overlap to determine enrichment or depletion beyond what is expected by chance. We assessed the significance of the overlap between the ST genes and cell cluster marker genes by calculating the *p* value using the hypergeometric cumulative distribution, with all genes serving as the background. Additionally, we determined the degree of cell cluster depletion by computing $-\log_{10}(1-p)$. In this study, there are the parameters for selecting differentially expressed genes from single-cell sequencing: scRNA_marker = scRNA _marker %>% filter (p_val_adj < 1e-05), scRNA_marker$d = scRNA_marker$pct.1 - scRNA_marker$pct.2, scRNA_marker = scRNA_marker %>% filter (d > 0.2), scRNA_marker = scRNA_marker %>% arrange (cluster,desc(avg_log2FC)) and spatial transcriptomic: region_marker = region_marker %>% filter (p_val_adj < 0.01), region_marker$d = region_marker$pct.1 - region_marker$pct.2, region_marker = region_marker %>% filter(d > 0.05), region_marker = region_marker %>% arrange(cluster,desc(avg_log2FC)).

Inferring CNV from single-Cell sequencing data

In order to differentiate malignant cells from normal cells, we utilized the R package inferCNV (version 1.8.0) to estimate copy number variation (CNV). Genes with low expression, defined as expression in less than 10 cells and a median expression value below 0.1, were filtered out. Initial CNVs for each region were estimated by inferCNV. We estimated the CNV for all cell clusters based on expression levels derived from single-cell sequencing data. The parameters used were --cutoff 0 and –noise_filter 0.2. To analyze gene expression within each sample, we standardized the expression values of cells and restricted them to a range of -1 to 1. The CNV score for each cell was then calculated as the quadratic sum of CNV regions.

Single-cell trajectory analysis

Data collected from Seurat can be easily imported into R package Monocle (version 2.20.0). The R package Monocle was applied to estimate the developmental pseudotime of all glioma cells. Initially, we filtered out genes with expression levels below 0.5 and those expressed in fewer than 200 cells. Using highly variable genes with a *p* value of $1e^{-40}$, we reordered the dataset according to different gene expression patterns. Subsequently, we utilized the DDRtree algorithm for dimensional reduction. Finally, we conducted pseudotime analysis to identify significant genes and their trajectories. Moreover, we employed the Branch Expression Analysis Modeling (BEAM) test to identify differential expression levels at a branch-specific level. Furthermore, we estimated the unsupervised inference of developmental directions of tumor cells using the VECTOR algorithm. In summary, we treated all tSNE dimensions related to tumor cells as an image, which was then divided into pixels. We generated the largest connected pixel network by linking adjacent pixels in the tSNE plot to infer the developmental direction.

SCENIC

Activated regulons in different tumor clusters were identified using R package SCENIC (version 1.3.1). Firstly, we input the expression matrix from single-cell sequencing, along with the corresponding transcription factor database of human (https://resources.aertslab.org/cistarget/). We used the GENIE3 algorithm with the following parameters: treeMethod = "RF", K = "sqrt", and nTrees = 1000, to calculate co-expression activity based on the raw count matrix input, using Spearman correlation. The filtered target genes were subsequently analyzed for motif enrichment using RcisTarget. To estimate regulatory activity scores for gene motifs located within 500 bp upstream and 10 kb around the TSS, we used an AUCell with a Wilcoxon rank-sum test. Last, important regulons modulated by key TFs were identified.

Predicting intercellular communication

To investigate the potential cell-cell communication in our single-cell transcriptomic data, we utilized the Cellchat (version 1.5.0) package, which is a computational tool for predicting and analyzing ligand-receptor interactions between different clusters. We performed ligand-receptor interaction analysis on all glioma cells and M2 macrophages using the CellChat package with default parameters. We used the CellChatDB.human database for ligand-receptor interaction analysis. The output of the analysis included a list of potential ligand-receptor pairs between glioma cells and M2 macrophages, as well as statistical significance scores for each interaction. We considered ligand-receptor pairs with a $p < 0.05$ to be statistically significant in our analysis.

Gene set enrichment analysis GSEA and pathway analysis

We conducted GSEA (Gene Set Enrichment Analysis) and pathway enrichment analysis on the top 200 marker genes in cluster 6, as well as the top 200 genes obtained using the differentialGeneTest function in Monocle using R package clusterProfiler (version 4.0.5) and David (https://david.ncifcrf.gov/). Gene signatures from Hallmark, GO and KEGG datasets were used as inputs to evaluate the pathway activity of different clusters. In single-cell sequencing, pathway signature scores were calculated using the AddModuleScore function.

Immune cell infiltration proportions in bulk-RNA level

To investigate the degree of immune cell infiltration in our bulk RNA sequencing data, we utilized the CIBERSORT function in R, which is a commonly used computational method for immune cell profiling. CIBERSORT uses gene expression data to predict the proportions of different immune cell types in a sample based on a pre-defined reference gene expression signature matrix. This allowed us to quantify the abundance of 22 distinct immune cell types within each sample. To perform the CIBERSORT analysis, we first preprocessed our raw RNA sequencing data and obtained gene expression profiles for each sample. We then ran the CIBERSORT algorithm using the

default parameters and the LM22 signature matrix, which was developed based on the gene expression profiles of purified immune cells. The output of the analysis included the predicted proportions of each immune cell type in each sample, as well as a CIBERSORT p value, which indicated the confidence level of the prediction. To ensure the accuracy of our results, we only included samples with a CIBERSORT $p < 0.05$ in our downstream analysis.

Survival analysis

We obtained glioma expression data from both the TCGA and Rembrandt datasets, which were accessed through the Gliovis platform. The expression of each gene is formatted as Log_2(TPM+1) scale with or without normalization. We performed survival analysis using the survival (version 3.2) package in R, fitting Kaplan-Meier survival curves with 50-50 percentiles. To visualize the survival plots, we utilized the ggsurvplot function from the survminer (version 0.4.9) package.

Correlation analysis

After downloading the expression data from GlioVis for the 20 different databases, we performed a Pearson correlation test between the expression levels of CEBPB and SPP1, across the different databases. If the Spearman correlation coefficient $r < 0.4$ and the $p < 0.01$, we considered the expression levels of CEBPB and SPP1 to be positively correlated in the database.
